# Urban Versus Lake Impacts on Heat Stress and Its Disparities in a Shoreline City

**DOI:** 10.1029/2023GH000869

**Published:** 2023-11-22

**Authors:** TC. Chakraborty, Jiali Wang, Yun Qian, William Pringle, Zhao Yang, Pengfei Xue

**Affiliations:** ^1^ Atmospheric, Climate, and Earth Sciences Division Pacific Northwest National Laboratory Richland WA USA; ^2^ Environmental Science Division Argonne National Laboratory Lemont IL USA; ^3^ Department of Civil, Environmental and Geospatial Engineering Michigan Technological University Houghton MI USA

**Keywords:** heat stress, urban climate, humidity, crowdsourced data, remote sensing, numerical modeling

## Abstract

Shoreline cities are influenced by both urban‐scale processes and land‐water interactions, with consequences on heat exposure and its disparities. Heat exposure studies over these cities have focused on air and skin temperature, even though moisture advection from water bodies can also modulate heat stress. Here, using an ensemble of model simulations covering Chicago, we find that Lake Michigan strongly reduces heat exposure (2.75°C reduction in maximum average air temperature in Chicago) and heat stress (maximum average wet bulb globe temperature reduced by 0.86°C) during the day, while urbanization enhances them at night (2.75 and 1.57°C increases in minimum average air and wet bulb globe temperature, respectively). We also demonstrate that urban and lake impacts on temperature (particularly skin temperature), including their extremes, and lake‐to‐land gradients, are stronger than the corresponding impacts on heat stress, partly due to humidity‐related feedback. Likewise, environmental disparities across community areas in Chicago seen for skin temperature are much higher (1.29°C increase for maximum average values per $10,000 higher median income per capita) than disparities in air temperature (0.50°C increase) and wet bulb globe temperature (0.23°C increase). The results call for consistent use of physiologically relevant heat exposure metrics to accurately capture the public health implications of urbanization.

## Key Points

- Lake Michigan reduces daytime heat stress in Chicago, while urbanization primarily increases nighttime heat stress
- Urban and lake impacts on skin temperature are stronger than the corresponding impacts on air and wet bulb globe temperature
- Disparities in skin temperature across Chicago’s community areas are higher than disparities in air and wet bulb globe temperature

## 1 Introduction

Due to increased urbanization adjacent to water bodies (Tibbetts, 2002), studies have frequently examined the complex interactions along the land‐water interface and their impacts on urban weather and climate (Hu & Xue, 2016; Keeler & Kristovich, 2012; Sharma et al., 2018; Shepherd & Burian, 2003; Theeuwes et al., 2013). The thermostatic effect of water leads to a positive thermal gradient from water bodies to land during daytime since the land warms faster than the water, generating lake or sea breezes that flow from over the cooler water surface to the warmer and lower pressure land surface (Birch et al., 2015; S. T. K. Miller et al., 2003). The effect reverses at night with the land near water bodies being warmer (due to higher thermal inertia of water) than land in the interior and land breezes generated from the land toward the water (J. Yang et al., 2022). These interactions set up strong temperature gradients across the waterfront that can influence heat exposure for coastal and shoreline populations. Several studies have examined this coastal influence on urban heat exposure using air temperature or satellite‐derived surface or skin temperature (Roth et al., 1989; Wu et al., 2019; J. Yang et al., 2022). Although skin and air temperature are coupled at large scales, pedestrians are exposed to ambient air temperature, not skin temperature, especially relevant within cities (Venter et al., 2021). Moreover, the physiological response to heat depends not only on air temperature, but also on relative humidity and wind speed (among other factors) (Anderson et al., 2013). For instance, all else remaining constant, more moisture in the air usually increases heat stress, which reduces the differential between human skin and core temperature; and thus the body's cooling efficiency (Im et al., 2017). Both urbanization and proximity to water bodies can modify ambient relative humidity and wind patterns. Urbanization can dry the air by modifying the city's surface energy balance, often referred to as the urban moisture or dry island effect (Chakraborty et al., 2022; X. Huang et al., 2021; Z. Wang et al., 2021), and impede wind flow by increasing surface roughness (Qian et al., 2022). On the other hand, breezes originating from water bodies can bring in more moisture during the day (S. T. K. Miller et al., 2003). Although previous urban modeling studies have considered physiologically relevant heat stress metric (Chakraborty, Newman, et al., 2023; K. Huang et al., 2021; Oleson et al., 2015; Zhao, 2021), they are generally not run with configurations or resolutions capable of resolving coastal gradients. Thus, there is a need to understand how the interaction between urbanization and adjacent water bodies impact air temperature, relative humidity, and wind, and thus heat stress, in shoreline cities.

Due to the heterogeneity of cities, heat exposure shows large intra‐urban variability. Across a global subset of cities (Chakraborty et al., 2019), and particularly in the US (Hsu et al., 2021), this variability leads to disproportionately higher heat exposure for poorer and other vulnerable communities. Some of these disparities relate to population dynamics within cities, frequently associated with historical discriminatory practices (Hoffman et al., 2020) as well as present inequities (Chakraborty, Newman, et al., 2023; Juday, 2015). Most multi‐city studies that have examined disparities in heat exposure have done so using satellite‐derived skin temperature (Benz & Burney, 2021; Chakraborty et al., 2019; Hoffman et al., 2020; Hsu et al., 2021). For land‐locked cities, these disparities are strongly associated with inequities in urban vegetation, with richer populations living in greener areas (Chakraborty et al., 2020). However, cities along the waterfront are more complicated due to competing effects of neighborhood greenness and access to waterfronts on real estate prices (N. G. Miller et al., 2019), and thus on population distributions. Regarding the humidity contribution to heat stress, the moisture brought in by sea and lake breezes into neighborhoods can be a strong function of their proximity to the waterfront, while neighborhood‐scale built‐up properties (including urban vegetation) would also impact near‐surface moisture content (Chakraborty et al., 2022; Qian et al., 2022). Although these complexities (the role of proximity to the water vs. neighborhood characteristics) are evident when examining satellite‐derived skin temperature (Chakraborty et al., 2020), we do not know the bulk outcome of population distributions and meteorological variability within shoreline cities on potential disparities in heat stress.

**Figure 1:**
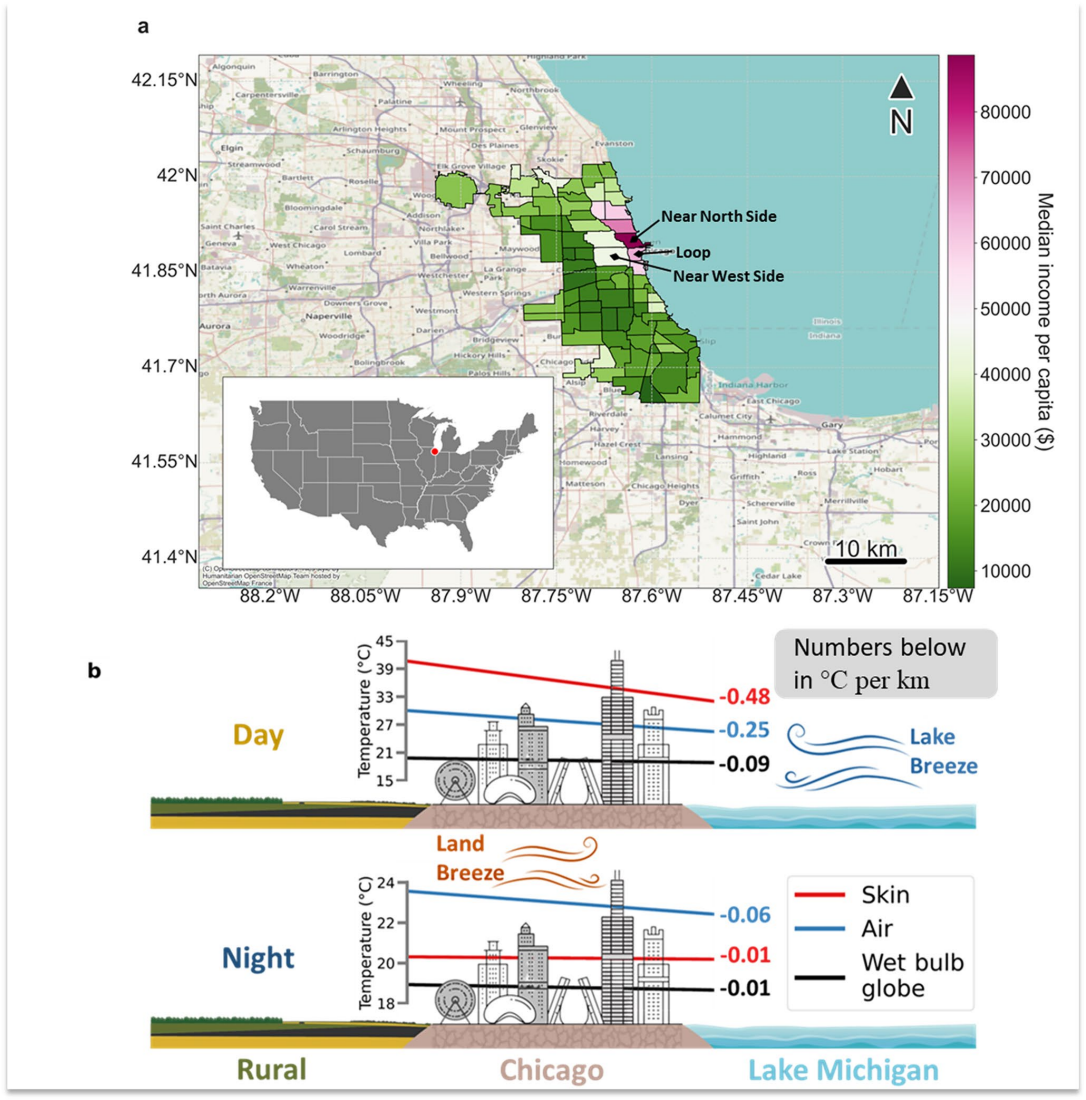
Study area and overview schematic. (a) Spatial extent of Chicago neighborhoods (inset marks location of Chicago within the United States) and the median income per capita in each. (b) Conceptual schematic of Chicago in reference to Lake Michigan and rural Illinois with daytime and nighttime (corresponding to MODIS Aqua overpasses) lake‐to‐land gradients in skin temperature, air temperature, and wet bulb globe temperature derived from the five‐member ensemble mean of WRF models simulations and the proximity of the neighborhood centroids to the lake shore. The numbers annotated with the colored gradients show the slopes of the best fit lines in °C per km distance from Lake Michigan. Note that the *x*‐axis is reversed here (compared to Figure 4) for consistency with the schematic. The representation of Chicago is purely illustrative and not to scale.

Here, we conduct several sensitivity experiments using the Weather Research and Forecasting (WRF) model with multi‐layer urban canopy representation and daily satellite‐derived boundary conditions for lake surface temperature. These simulations are used to isolate the urban versus lake effect on the spatial variability of heat‐related extremes within 77 community areas (neighborhoods henceforth) of Chicago during a typical summer (of 2018). We choose Chicago since it is a shoreline city (third largest among all US cities) to the west of Lake Michigan with a history of heat‐related weather extremes (Conry et al., 2015; Kunkel et al., 1996). Previous studies have shown that the daytime breeze originating from Lake Michigan can penetrate 15–30 km inland, moving cooler and more humid air into Chicago during daytime, with an overall reduction in heat extremes (Conry et al., 2015; Keeler & Kristovich, 2012; Kunkel et al., 1996). However, these studies, and others that have investigated heat exposure in various shoreline cities (Roth et al., 1989; Wu et al., 2019; J. Yang et al., 2022), have mainly focused on skin or air temperature. Here, with the intent of focusing on physiologically relevant heat extremes, we also consider wet bulb globe temperature, the International Organization for Standardization (ISO) standard for occupational heat stress (Iso, 2017) that considers the combined impact of air temperature, relative humidity, wind speed, and solar radiation exposure on human health (Heo et al., 2019). The neighborhoods in Chicago show large residential segregation, with the richest neighborhoods lying along the waterfront (Figure 1a). Thus, we also examine disparities in heat stress and heat exposure across these neighborhoods and how they are impacted by the lake‐to‐land gradients. Our results show that although the lake and urbanization distinctly impact the magnitudes and gradients of air and skin temperature, these impacts flatten out for wet bulb globe temperature. The overall variability of maximum and minimum wet bulb globe temperature within Chicago is strongly controlled by air temperature, though wind speed and relative humidity also play detectable roles. Furthermore, heat exposure disparities and their extremes are generally more prominent when using skin temperature instead of air and wet bulb globe temperature, indicating the importance of using physiologically relevant variables when quantifying these environmental inequities.

## 2 Methods

### 2.1 Regions of Interest and Socioeconomic Data

Chicago is the third largest city in the United States, housing around 2.7 million people, and located on the southwestern side of Lake Michigan (Figure 1a). The city is affected by large pressure gradients during the summer due to the differential heating over the city and over Lake Michigan, resulting in strong lake breeze during the day (Laird et al., 2001). To examine spatial variability across this city, we consider the 77 community areas, which are commonly used to provide statistical summaries and for urban planning (Figure 1a). Each community area includes estimates of median income per capita (corresponding to U.S. census data for 2008–2012), which we extract from the City of Chicago's data portal (https://data.cityofchicago.org/). Since income is not a comprehensive proxy for vulnerable populations, we also consider the Hardship index, a more relevant metric of socioeconomic vulnerability that includes contributions from six factors—namely unemployment, lack of secondary education, per capita income, percentage population below poverty level, overcrowded housing, and age dependency—after normalization (Amdat, 2021). Overall, both Hardship index and median income show similar spatial variability, with northern lakefront neighborhoods showing highest median income per capita and lowest Hardship index (Figure 1a; Figure S4a in Supporting Information S1).

### 2.2 Model Simulations

We use Weather Research and Forecasting (WRF; version 4.3.1) model simulations (Skamarock & Klemp, 2008) centered at 45.5°N and 85.0°W and covering the Great Lakes region with a grid spacing of 4 km and 50 stretched vertical levels starting from 30.2 m above the ground (J. Wang et al., 2022) to simulate summertime conditions in Chicago. The initial and boundary conditions for the model (at 0.25° resolution every 3 hr) are taken from the European Centre for Medium‐Range Weather Forecasts atmospheric reanalysis of the global climate, version 5 (ERA5) (Hersbach et al., 2020), while the boundary condition for the lake surface temperature (daily at 1.3 km) is from satellite‐derived estimates from the National Oceanic and Atmospheric Administration (NOAA) Great Lakes Surface Environmental Analysis (Schwab et al., 1992). The model is run with the Rapid Radiative Transfer Model (RRTM) (Iacono et al., 2008) for shortwave and longwave radiation, the Yonsei University (YSU) planetary boundary layer scheme (Hong & Lim, 2006), revised Atmospheric Research Mesoscale Model Monin‐Obukhov surface layer scheme (Jiménez et al., 2012), Thompson microphysics (Thompson et al., 2004), and the Unified Noah land surface model (Noah LSM) (F. Chen & Dudhia, 2001).

We run the model in three separate configurations: one corresponding to the present‐day conditions (control), and two counterfactual scenarios with Lake Michigan (no lake) and Chicago urban grids (no urban) removed, respectively. For the control simulation, we use the latest multilayer urban canopy model (MLUCM) in WRF with Building Effect Parameterization (BEP) (Martilli et al., 2002) and Building Energy Model (BEM) (Salamanca et al., 2010). Together, BEP and BEM can represent the building impact on airflow and the exchange of energy between buildings and their surroundings. Grid‐wise land cover is provided to the model from Moderate Resolution Imaging Spectroradiometer (MODIS) land cover with 21 classes, which WRF uses by default. The urban grids correspond to the high‐density class of the MLUCM and have a fixed built‐up fraction of 0.9. To examine the impact of urbanization and lake, respectively, the urban Chicago and Lake Michigan grids are replaced by cropland, the dominant land cover in the region. Thus, the difference between the control and no urban simulation provides the local urban effect, while the difference between the control and no lake simulation gives us the effect of Lake Michigan. This replacement of one surface type with another to examine the impacts on climate is a common approach in numerical modeling (K. Huang et al., 2021; Sarangi et al., 2021; Theeuwes et al., 2013). In contrast, for observational studies, such as those on urban heat islands, where a similar replacement of surface type is not methodologically possible, space‐for‐time substitution approaches are used instead, generally by defining buffers surrounding the urban area to serve as a rural reference (Q. Yang et al., 2023). This is because considering regions farther away (say, the other side of Lake Michigan) as a background reference would result in differences not just due to surface type, but also due to synoptic‐scale processes.

All the model configurations are run five times with different initializations 12 hr apart between 12 May 2018 and 14 May 2018 and the simulations are run till 1 September 2018 (J. Wang et al., 2023). The five different initializations form an ensemble and are meant to test the sensitivity of the results to initial conditions. The results for the 3 months of summer (June, July, August) are used in the study. The WRF outputs are at hourly resolution for each model grid.

### 2.3 Calculating Heat Indices

We estimate heat indices from the model simulations to represent human physiological response to heat extremes. First, we calculate wet‐bulb temperature, a thermodynamic measure of humidity in a parcel of air, which is often used as a proxy for heat stress in climate studies (Im et al., 2017; Raymond et al., 2020; Sherwood & Huber, 2010) using an iterative approach (Stipanuk, 1973). We also compute wet bulb globe temperature, which is the ISO (International Organization for Standardization) standard for occupational heat stress (Iso, 2017), using the following empirical equation (Ono & Tonouchi, 2014):

(1) $$\text{WBGT}=0.735\times \text{AT}+0.0374\times \text{RH}+0.00292\times \text{AT}\times \text{RH}+7.619\times \text{SR}-4.557\times {\text{SR}}^{2}\;-0.0572\times \text{WS}-4.064$$

where WBGT is in °C, SR is the solar insolation at the surface (in kW m^−2^), WS is wind speed at 10 m (in m s^−1^), RH is the 2‐m relative humidity (in %), and AT is the 2‐m air temperature (in °C). Wet‐bulb temperature generally has a stronger dependence on humidity than other heat stress metrics (Chakraborty et al., 2022), and is not strongly linked to health outcomes till physiological thresholds are reached (Sherwood, 2018). Moreover, these physiological thresholds assume no energy input (such as from solar radiation) into the human body and continuous gale force winds, which is quite unrealistic. Since urbanization and the lake both impact wind speed, and solar exposure is expected under most outdoor conditions during daytime, wet bulb globe temperature is a more complete indicator of outdoor heat stress (Heo et al., 2019), which we focus on when describing the results. The overall patterns found in the study are similar when using wet‐bulb temperature, with this variable showing even weaker associations with distance from the coast and income than wet bulb globe temperature (Figures S1, S3, and S4 in Supporting Information S1).

### 2.4 Data Processing at Neighborhood‐Scale

Average diurnal cycles are generated for all relevant variables (air temperature, relative humidity, wet bulb globe temperature, wet‐bulb temperature, and skin temperature) from the hourly model outputs for summer 2018. From these cycles, the highest and lowest values are extracted for each grid, representing the maximum and minimum value for the average summer day in 2018, and summarized for each of the community areas. Similarly, the 95th and 98th percentile of these variables are also extracted from the hourly outputs and combined to create summaries for each geographic aggregation.

To check the consistency of patterns with observations, we also extract satellite data for summer 2018 over Chicago and summarize for the same regions. Satellite‐derived skin temperature (more commonly called land surface temperature; LST) is from 8‐day composite 1 km MODIS observations on board the Aqua satellite. Pixel‐level quality control flags are used to only keep data with an uncertainty of less than or equal to 3°C, following previous studies (Chakraborty et al., 2020). Note that this uncertainty relates to the algorithm used to estimate LST from MODIS observations in the thermal bands; and is different from the sources of uncertainty in the coupled model simulations (see Sections 2.7 and 3.6). The Aqua overpass for day and night are at around 1:30 p.m. and 1:30 a.m. local time, respectively, which is not the same as the time of the maximum and minimum average skin temperature. For a more accurate comparison, WRF‐simulated skin temperatures corresponding to these overpasses (1–2 p.m. local time for daytime; 1–2 a.m. for nighttime) are estimated from the control runs (Figure 1; Figures S1 and S2 in Supporting Information S1). We also calculate the normalized difference vegetation index (NDVI), a proxy of live green vegetation at the surface (Rouse et al., 1974), from MODIS surface reflectance product. The NDVI is calculated as:

(2) NDVI=(NIR−RED)/(NIR+RED)

where NIR is the reflectance in the near‐infrared and RED is the reflectance in the red band. Only the best quality pixels are selected before calculating the NDVI. For both skin temperature and NDVI, we also mask out all pixels corresponding to surface water (at 30 m resolution) from the Global Surface Water data set (Pekel et al., 2016) since the urban grids in WRF do not include any surface water fraction. All satellite remote sensing and geospatial data processing are done on the Google Earth Engine platform (Gorelick et al., 2017).

### 2.5 Factor Analysis Using Gridded Model Simulations

We examine the impact of different factors in Equation 1 on spatial variability of maximum and minimum average wet bulb globe temperature. For this, the factors (AT, RH, WS, and SR) corresponding to the time of these maximum and minimum average WBGT values are extracted for each grid overlaying Chicago from the control simulations. Then, multiple linear regressions are used to express wet bulb globe temperature (maximum or minimum average) as a function of its factors:

(3) $$\text{WBGT}=\beta_{0}+\beta_{1}\text{AT}+\beta_{2}\text{RH}+\beta_{3}\text{WS}+\beta_{4}\text{SR}$$

where *β*_0_ is the intercept and *β*_1_, *β*_2_, *β*_3_, and *β*_4_ are the regression coefficients of air temperature, relative humidity, wind speed, and solar radiation, respectively. These coefficients give the sensitivity of wet bulb globe temperature to a unit change in the associated factor, assuming other factors are held constant. Overall, the magnitude and direction of these coefficients give an estimate of the strength and direction of association between the factor and wet bulb globe temperature. Since the factors have different range of values, which would impact the regression coefficients, we provide a second perspective by also computing similar sensitivities after rescaling all factors to lie between 0 and 1. In this scaled case, the relative importance of factors estimated purely due to the unit used is accounted for. Note that this method assumes linear independence of the factors. In reality, some of these factors are not independent. For instance, increasing air temperature would reduce relative humidity even if the total moisture content of the air remains constant. This potential collinearity between the factors should be kept in mind when assessing the results. However, that air temperature is vastly more important for wet bulb globe temperature is seen in the regression coefficients found here (see Section 3) and also easily interpretable from the coefficients in Equation 1.

### 2.6 Extracting Crowdsourced Weather Data in Chicago

The model configuration used here have been evaluated against buoy measurements and in situ measurements throughout the Great Lakes Region in a previous study (J. Wang et al., 2023). However, in that study, the focus was more on day‐night patterns and overall accuracy rather than spatial variability within Chicago. Since urban areas rarely have standard weather stations, our primary evaluation of the modeled skin temperature uses satellite observations. However, with the intent of capturing both the magnitude and the spatial variability of other relevant variables important for heat stress, we also extract all citizen science weather station data in Chicago that have data covering the full period of summer, 2018. These data are measured by outdoor personal weather stations from Netatmo, a manufacturer of smart home devices (Venter et al., 2021). Although only five such stations exist within Chicago (Figure S3a in Supporting Information S1), they are more spread out than the airport weather station data normally used for such model evaluations (Tan et al., 2022). We calculate the maximum and minimum average air temperature and relative humidity from each of these stations for summer 2018 and examine associations between these values and the corresponding neighborhood‐level estimates from the five‐member ensemble mean of the WRF control simulations.

### 2.7 Model Evaluation and Uncertainties

Though we evaluate our WRF‐simulated variables against satellites observations after aggregating to the neighborhood scale (see below and Figures S1 and S2 in Supporting Information S1), against both buoy and in situ measurements to examine diurnal patterns (J. Wang et al., 2023), and against crowdsourced measurements to test for spatial variability (see below and Figure S3 in Supporting Information S1), it is important to stress the uncertainties in both our model estimates and the evaluations. On the modeling side, WRF cannot fully capture the spatial variability of a heterogenous city like Chicago (Qian et al., 2022). For instance, the urban density classes have fixed radiative and thermodynamic parameters. Moreover, street‐level urban vegetation is not explicitly represented, which can modulate wind speed, air temperature, and surface moisture budget, especially evapotranspiration and thus relative humidity (Krayenhoff et al., 2020). Urban hydrology is also poorly represented in WRF, with no real subsurface drainage, which would also impact relative humidity. On the evaluation side, while WRF‐simulated skin temperature is integrated over an idealized 3D urban canyon, satellites provide a 2D directional view of a 3D heterogeneous urban landscape for clear‐sky conditions (Du et al., 2023; Stewart et al., 2021). Thus, some differences in magnitude between skin temperatures from MODIS and WRF are expected, especially for grids with higher urban density. Finally, the citizen weather station measurements represent information about a much smaller footprint than the WRF grids.

With these uncertainties in mind, the WRF‐simulated and MODIS‐observed daytime estimates show a correlation of 0.29, mean bias error of −2.4°C, and a slope of the line of best fit close to unity (Figure S2a in Supporting Information S1). The impact of the vegetation representation in WRF is also evident during daytime, as the difference between modeled and observed skin temperature is positively correlated with normalized difference vegetation index (NDVI; Figure S2c in Supporting Information S1), which is a proxy for live green vegetation at the surface (Rouse et al., 1974). At night, however, the model cannot capture the variability of observed skin temperature across neighborhoods, showing a negative correlation with an *r* ^2^ value close to 0 (Figure S2b in Supporting Information S1). This discrepancy between modeled and observed nighttime skin temperature is also evident from the opposite direction of the lake‐to‐land gradient in Figure 4 and the opposite associations with median income (Figure 7b; Figure S8 in Supporting Information S1). In addition to differences caused by the satellite view of the 3D urban landscape providing a directional estimate of clear‐sky skin temperature (unlike the WRF simulated value, which is integrated over the urban facades and accounts for all sky conditions), we note that satellite‐derived values have a more continuous distribution than the simulated values (Figure S2a in Supporting Information S1). This makes sense since the skin temperature is strongly constrained by the surface energy budget, which, in turn, is constrained by the prescribed parameters of urban surfaces. These parameters being fixed in WRF (Chakraborty et al., 2021; Z.‐H. Wang et al., 2011), the spatial variability of simulated skin temperature is also similarly limited, leading to the distinct clusters seen in Figure S2a of the Supporting Information S1 during daytime. This issue is of less importance for air temperature, which we further evaluate below, since it is well‐mixed through horizontal advection.

While the sample size is small, for evaluations of simulated maximum and minimum average air temperature and relative humidity against weather station measurements, positive correlations are seen in all cases other than for minimum average air temperature (Figure S3 in Supporting Information S1). For air temperature, the mean bias errors are less than 0.5°C, while the errors are higher for relative humidity. Minimum average relative humidity, which is during daytime, is largely underestimated by WRF even though the general spatial pattern is captured. This is potentially also related to the lack of explicit vegetation representation and hydrology within the urban grids, which would impact the near‐surface relative humidity simulated by the model. In comparison, the personal weather stations are generally not set up over impervious surfaces, but rather in lawns and backyards, which would have higher relative humidity. Finally, moisture emissions from anthropogenic activities are also poorly represented in models (X. Huang et al., 2021; Z. Wang et al., 2021), which would also impact relative humidity. We stress that these evaluations are done more as a sanity check than to confirm the “truth” represented by the model simulations. See the relevant discussions about the uncertainties earlier. However, the model simulations are helpful for capturing the relative changes among the different variables (air temperature, skin temperature, wet‐bulb temperature, and wet bulb globe temperature) to perturbations like the presence of the lake or built‐up areas since many of the processes modulating these variables are explicitly simulated via the model physics.

## 3 Results

### 3.1 Urban Versus Lake Impacts on Heat Stress Across Neighborhoods

**Figure 2:**
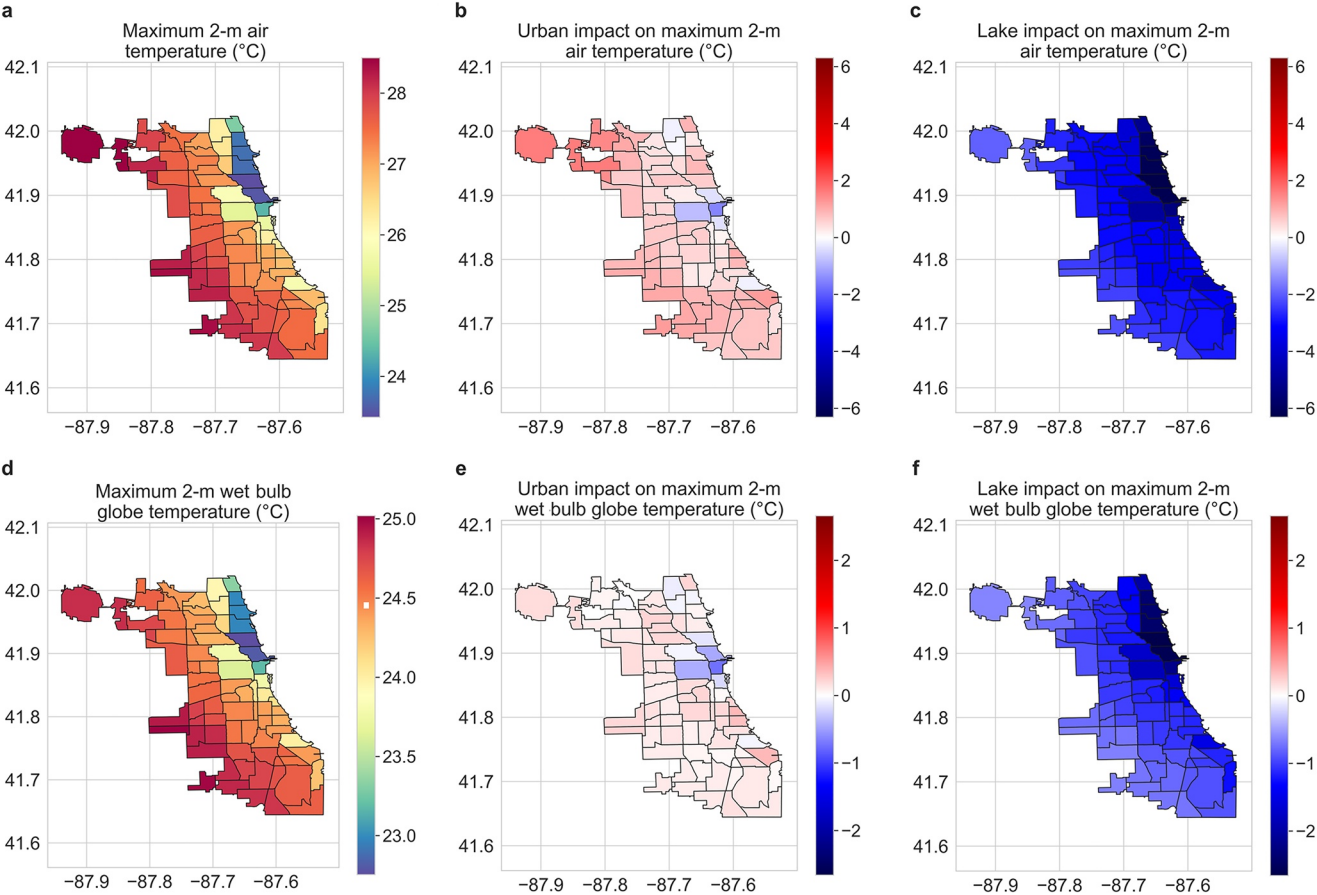
Urban and lake impacts on maximum air and wet‐bulb globe temperature. Neighborhood‐scale summaries of WRF simulated maximum average (a) air temperature and (d) wet‐bulb globe temperature for summer 2018 in Chicago. (b and e) Urban impact on maximum average air temperature and wet‐bulb globe temperature, respectively. (c and f) Lake impact on those variables.

**Figure 3:**
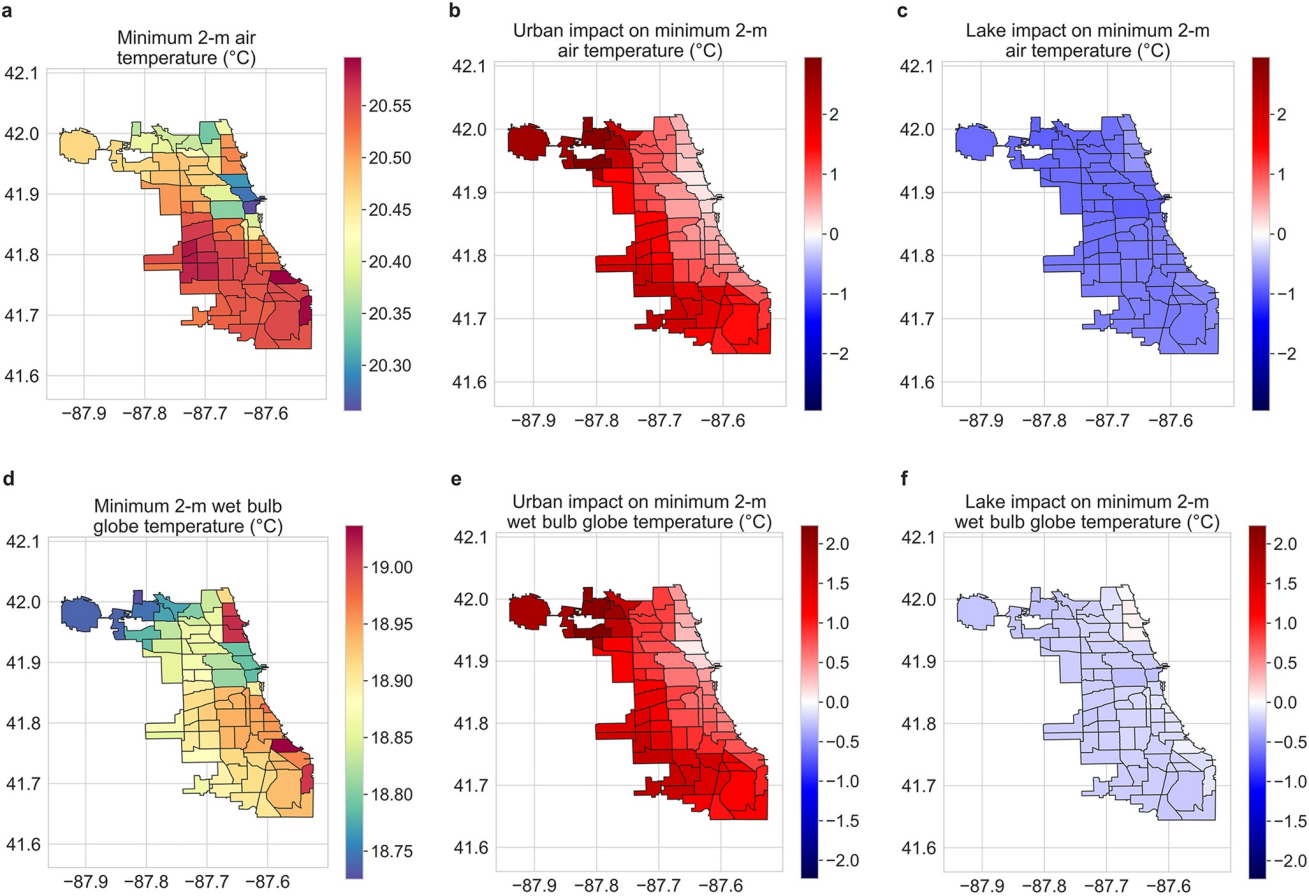
Urban and lake impacts on minimum air and wet‐bulb globe temperature. Neighborhood‐scale summaries of WRF simulated minimum average (a) air temperature and (d) wet‐bulb globe temperature for summer 2018 in Chicago. (b and e) Urban impact on minimum average air temperature and wet‐bulb globe temperature, respectively. (c and f) Lake impact on those variables.

Disaggregating the WRF model simulations (control runs that include both urban and lake grids) into five‐member ensemble mean summaries for Chicago neighborhoods, we find a lake‐to‐land gradient in skin temperature, air temperature, and relative humidity, particularly during daytime (Figures 1, 2, 3; Figures S4 and S5 in Supporting Information S1). For instance, summertime maximum average air temperature and minimum average relative humidity, both during daytime, increase and decrease, respectively, as we move away from the lake shore (Figure 2a; Figure S5d in Supporting Information S1). Maximum average relative humidity also shows a lake‐to‐land gradient, though minimum average air temperature has a weaker gradient (Figure 2a). Wet bulb globe temperature also shows a similar but weaker lake‐to‐land gradient (Figure 1b).

The urban and lake impacts on these variables are estimated through perturbation simulations where the built‐up and lake grids are replaced with cropland (see Section 2). With some exceptions, air temperature and wet bulb globe temperature increase due to urbanization across neighborhoods. The urban impact is particularly evident at night for these variables. The stronger urban impact on minimum air temperature compared to maximum is in line with modeling and observational estimates across cities (Qian et al., 2022; Sarangi et al., 2021; Venter et al., 2021). Urbanization appears to slightly reduce maximum average air and wet bulb globe temperature in the Near West Side, Near North Side, and the Loop neighborhoods (Figures 1, 2, and 2e). Urbanization also shows mixed impact on skin temperature according to the WRF simulations (Figures S4b and S5b in Supporting Information S1). Here, it should be noted that the surface of a multi‐layer urban canopy is fundamentally different from that of a cropland grid, which is treated as a flat slab in the land‐surface model, which could lead to some of these discrepancies. Finally, urbanization also reduces maximum and minimum relative humidity across most neighborhoods (Figures S1d and S5d in Supporting Information S1). The impact of Lake Michigan on the micro‐climate of Chicago neighborhoods is more consistent, with general decreases in maximum and minimum temperature and heat indices (but with different magnitudes; see Section 3.2) and increases in relative humidity (Figures 2 and 3; Figures S4 and S5 in Supporting Information S1).

### 3.2 Lake‐To‐Land Gradients in Different Measures of Heat Exposure

**Figure 4:**
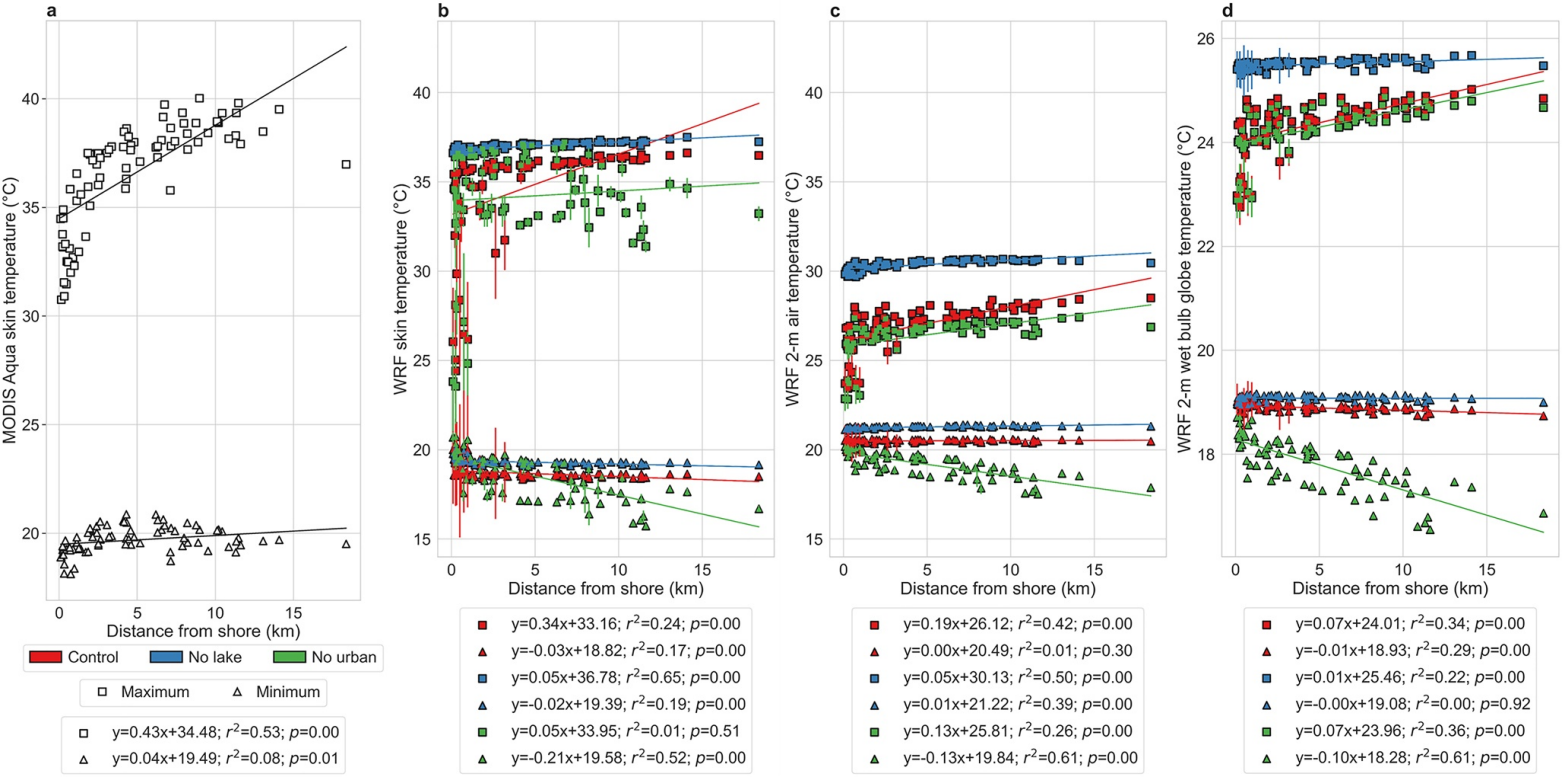
Lake‐to‐land gradients in measures of heat exposure. Associations between (a) MODIS Aqua daytime and nighttime skin temperature (more accurately, land surface temperature) and WRF simulated maximum and minimum average (b) skin temperature, (c) air temperature, and (d) wet bulb globe temperature in summer 2018 for 77 neighborhoods in Chicago and the corresponding distance of the centroid of each neighborhood to the lake shore. The points represent the mean values from the five members of the ensemble and the error bars correspond to standard errors across the members. The lines of best fit are shown and the associated equations, coefficients of determination, and *p* values are in the legend. The square symbols show the maximum averages (daytime for MODIS) and the triangles are for the minimum averages (nighttime for MODIS). The three colors (red, blue, and green) show the results for control, no lake, and no urban simulations, respectively.

To quantitatively assess the lake‐to‐land gradients, visually evident in Figure 2, we examine associations between the variables for each neighborhood and the distance of the centroid of the neighborhood from the lake shore (Figure 4). This distance is a bulk proxy of the lake effect, including the impact of horizontal exchange of heat and moisture via lake and land breezes. We also compare the lake‐to‐land gradients of the modeled variables (Figure 1b) with corresponding gradients of skin temperature measured by the Moderate Resolution Imaging Spectroradiometer (MODIS) sensor aboard NASA's Aqua satellite (Figure 4a). In Figure 4, the maximum and minimum values for the satellite estimates correspond to the satellite overpass times for daytime and nighttime (∼1:30 p.m. and ∼1:30 a.m. local time equatorial overpass), respectively, while in WRF, they are the actual modeled extrema (see Section 2). Although these associations can be non‐linear, with stronger gradients seen at the land‐water interface, we use linear models for easier interpretability of the results.

Overall, the maximum skin temperature in both the control model simulation and the MODIS observations show positive correlations with the distance from the shore with similar slopes (0.43 vs. 0.34; Figures 4a and 4b; all in °C per km), though the coefficient of determination is weaker for WRF (*r* ^2^ = 0.24 vs. 0.53 for MODIS). Note that these slopes are even closer (0.43 vs. 0.48; see Figure 1b) when WRF simulations corresponding to the MODIS overpass times are used (Figure 1b). For minimum (∼1:30 a.m. for MODIS) skin temperature, the data are generally uncorrelated. Although the maximum air and wet bulb globe temperatures also show positive slopes with the distance from the shore, the sensitivity decreases from skin to air to wet bulb globe temperature (0.34, 0.19, and 0.07, respectively).

The counterfactual scenario where the Chicago urban grids are removed has a large impact on the gradient of skin temperature (0.34–0.05), a smaller impact on air temperature (0.19–0.13), and practically no impact on wet bulb globe temperature (0.07 for both and similar offsets; Figure 4d). The sign of the slope flips to positive from negative for maximum relative humidity. During nighttime (i.e., minimum values for all variables and maximum for relative humidity), the impact of urbanization is more evident, with the lake‐to‐land gradient strengthening for all cases. This suggests that the presence of Chicago counteracts the naturally expected lake‐to‐land gradient during nighttime. For the counterfactual scenario with no Lake Michigan, little impact is seen on minimum average variables compared to the control runs, but we see large offsets during daytime, with increases in air temperature and wet bulb globe temperature. Overall, it is evident that Lake Michigan has a stronger impact on heat exposure and heat stress in the region during daytime, while urbanization has a stronger impact at night. For almost all variables, the standard errors across the five ensemble members are greatest closest to the shore (Figure 4; Figure S6 in Supporting Information S1), demonstrating the high sensitivity of the micro‐climate at the land‐water interface simulated by the model to initial conditions (J. Wang et al., 2022).

### 3.3 Urban and Lake Impacts on Extremes Versus the Summer Climatological Mean

The previous analysis is indicative of the different sensitivities of the different measures of heat exposure and heat stress (air temperature and wet bulb globe temperature) to the distance from the water. However, these variables have different standard ranges, making the slopes not directly comparable. So, we calculate the absolute and percentage changes in the relevant variables due to urbanization and the lake within Chicago. Overall, the results seen in the sensitivity analysis are confirmed here (Table 1). Both percentage and absolute changes are higher for maximum average skin temperature than for maximum average air temperature during daytime and these changes are higher still than wet bulb globe temperature. The higher impact of urbanization on minimum average air temperature (2.02°C; compared to 0.99°C for maximum) and maximum average skin temperature (2.55°C; compared to 1.24°C for minimum) is also captured. The higher urban impact on daytime skin temperature partly relates to the lack of evaporative dissipation of heat over pervious surfaces (Paschalis et al., 2021), while this additional stored heat gets gradually released at night into the near‐surface air (Zhao et al., 2014). Finally, Lake Michigan generally has a stronger impact on the measures of heat exposure during daytime (up to 9% reduction for maximum average air temperature) and weaker effects at night (less than 4% decrease in all cases).

**Table 1:** Impacts of Urbanization and Lake Michigan Within Chicago

	Skin temperature (°C)	Air temperature (°C)	Wet bulb globe temperature (°C)	Relative humidity (%)
	Summer composite maximum			
Urban Effect	2.55 (7.6%)	0.99 (3.7%)	0.08 (0.33%)	−7.93 (−9.13%)
Lake Effect	−0.98 (−2.63%)	−2.75 (−9%)	−0.86 (−3.39%)	5.66 (7.72%)
	Summer composite minimum			
Urban Effect	1.24 (7.17%)	2.02 (10.96%)	1.57 (9.1%)	−7.23 (−13.9%)
Lake Effect	−0.68 (−3.56%)	−0.81 (−3.82%)	−0.23 (−1.2%)	6.27 (15.08%)
	Summer 98th percentile			
Urban Effect	2.86 (7.09%)	1.84 (5.45%)	0.06 (0.2%)	−3.2 (−3.29%)
Lake Effect	−0.78 (−1.77%)	0.36 (−1%)	−0.12 (−0.41%)	1.91 (2.08%)
	Summer 95th percentile			
Urban Effect	2.34 (6.13%)	2.29 (7.18%)	0.23 (0.81%)	−3.42 (−3.62%)
Lake Effect	−0.41 (−3.32%)	−0.58 (−1.66%)	−0.26 (−0.89%)	2.91 (3.3%)

*Note*. Summary of urban and lake effect on maximum and minimum averages and the 95th and 98th percentiles of skin temperature, air temperature, wet bulb globe temperature, and relative humidity in summer 2018 based on the five‐member ensemble mean of WRF models simulations.

We also examine changes (absolute and percentage) in the 95th and 98th percentile of these variables (from all hourly data) for the summer of 2018 due to the lake and urbanization (Table 1). Although the urban impact on the 95th and 98th percentile of air temperature is higher than that for the maximum average, the changes in wet bulb globe temperature extremes are lower. This is possible because as the air warms, all else remaining constant, the relative humidity will decrease, thus moderating heat stress. Considering the whole of Chicago, the 95th and 98th percentiles of wet bulb globe temperature (28.6 and 29.8°C, respectively) exceed the high risk threshold (28°C) used by the U.S. military (Willett & Sherwood, 2012). However, we find a low impact of urbanization on summer heat stress extremes (upper bound increase of 0.23°C for the 95th percentile of summer wet bulb globe temperature). Finally, both urbanization and the lake have smaller effects on the 98th percentile of heat exposure and heat stress than on the 95th percentiles, suggesting a flattening of the upper tail of their distributions. It is important to note that our goal here was to examine the climatological extremes for these variables during a typical summer. Individual extreme heat events are usually much shorter (Tan et al., 2022) and influenced by regional and continental‐scale synoptic processes during or before those periods.

### 3.4 Changes in Other Factors and Contributors to Heat Stress

**Figure 5:**
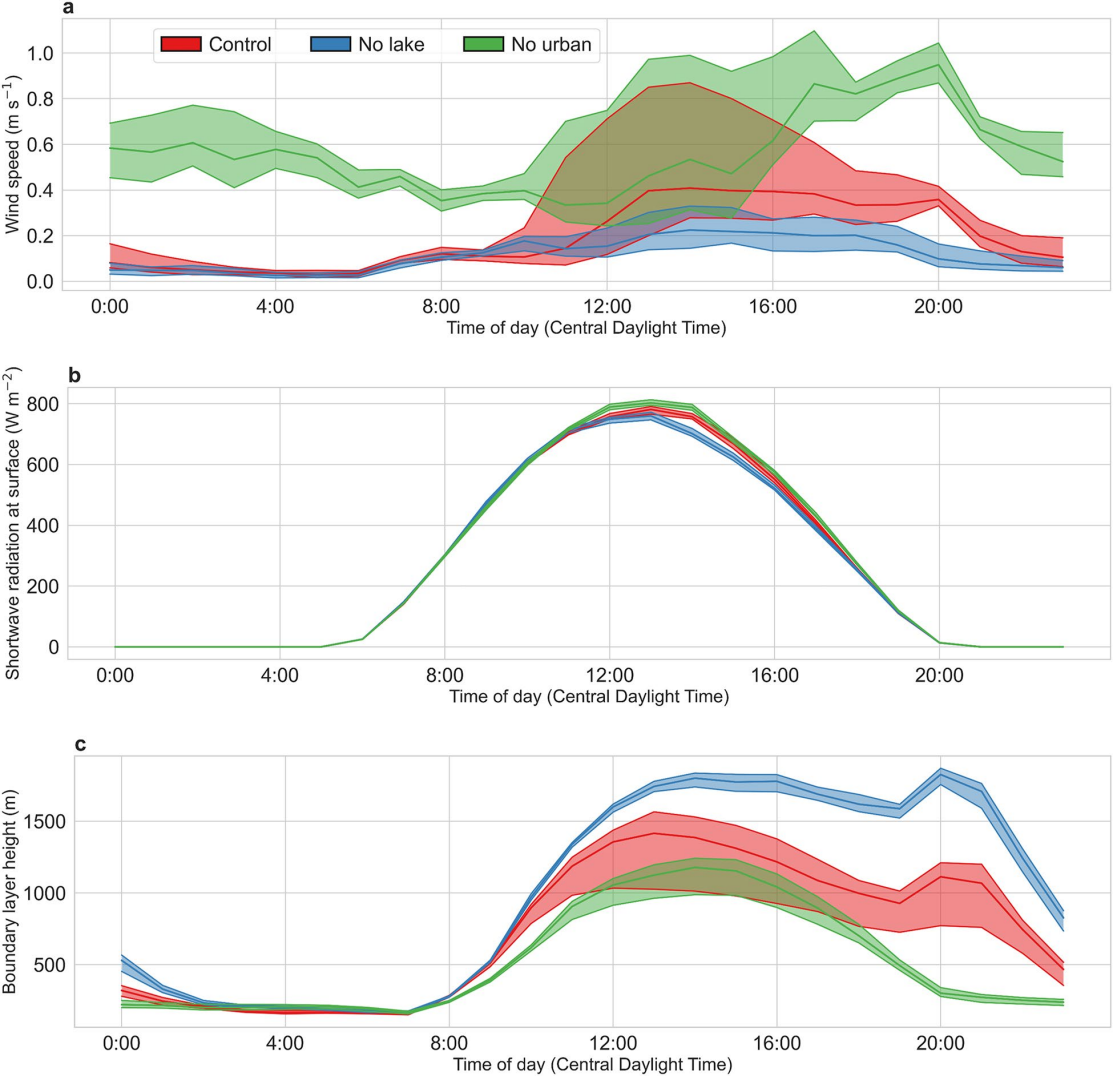
Diurnal variability of other relevant factors. Diurnal composites of (a) wind speed, (b) incoming shortwave radiation, and c boundary layer height in summer 2018 across model grids overlaying Chicago. The upper and lower lines represent the 75% and 25% percentile (across grids) of the mean values, and the middle line is for the median, all by hour of the day. Results are shown for the first member of the ensemble of WRF simulations.

In addition to air temperature and relative humidity, wet bulb globe temperature is also a function of wind speed and solar insolation (see Equation 1 in Section 2). Urbanization can impact both of these variables. We examine diurnal median composites of wind speed and the shortwave radiation incident at the surface over the city from one of the members of the ensemble (Figure 5). As expected, wind speed peaks during daytime, with large spread across the grids overlaying Chicago, while nights are consistently calmer across these grids (Figure 5a). Overall, removing the urban grids increases median wind speed, particularly at night, which is expected due to the decreased surface roughness (from urban structures to cropland) (Qian et al., 2022; Zhao et al., 2014). During daytime, wind speed changes are less. This does not mean that the urban impacts are less. Instead, it is because the land‐to‐lake temperature gradients are decreased, which weakens the lake breeze. This effect partly compensates for the impeding effects of urban roughness. The role of both roughness and thermal gradients is also evident from the almost uniformly low wind speed throughout the day in the no lake simulation, when urban roughness effects are still active, and the lake (or rather, the croplands where the lake would have been)‐to‐land temperature differences are small (Figure 4b).

Incoming shortwave does not vary much across grids and shows minor changes across these simulations, with lowest value in the no lake run (Figure 5b). This is probably because of changes in cloud cover in the three simulations influenced by changes in surface properties and moisture fluxes (J. Wang et al., 2022). We also look at the diurnal evolution of the boundary layer height across these grids for the three simulations (Figure 5c). This height is a proxy for the near‐surface stability and overall convective efficiency over the city. Overall, we see low boundary layer height during stable nights, with large increases during daytime as the atmosphere becomes unstable. The boundary layer is highest in the no lake simulation, the inverse of what is seen for wind speed. An interesting feature is the growth of boundary layer after around 7 p.m. local time in both simulations with the multilayer urban canopy model. This is a consequence of the sensitivity of this boundary layer scheme to the prescribed addition of anthropogenic flux during that time. This bump disappears when a different boundary layer scheme is used (not shown).

**Figure 6:**
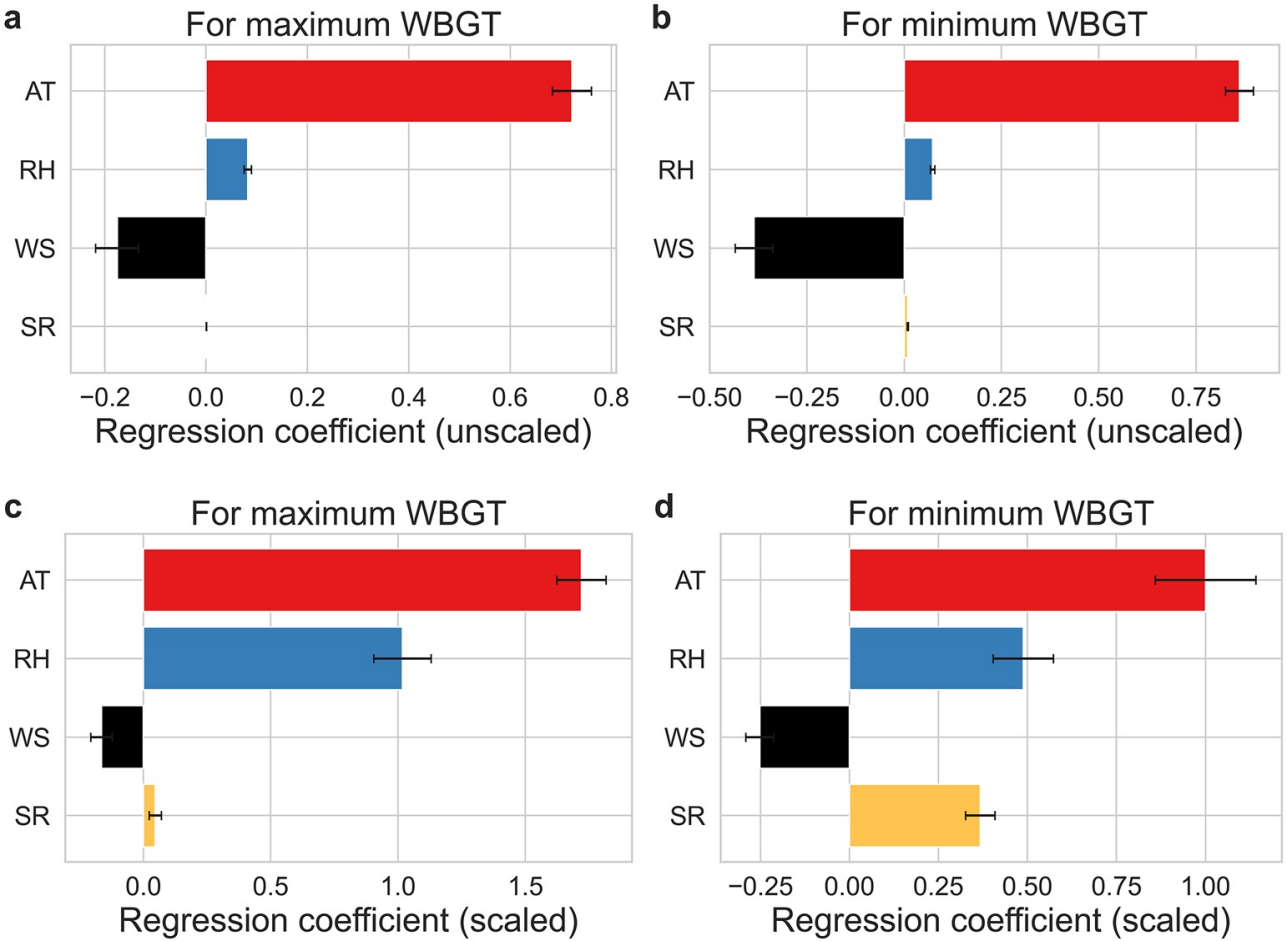
Sensitivity of wet bulb globe temperature to factors. Regression coefficients for air temperature (AT), relative humidity (RH), wind speed (WS), and incoming shortwave radiation (SR) from multiple linear regressions that explain spatial variability of (a) maximum and (b) minimum wet bulb globe temperature (WBGT) over the model grid overlaying Chicago. The error bars show the standard error across the five members of the ensemble. (c and d) are similar to a and b, but from regressions where all variables have been scaled to lie between 0 and 1.

Using multiple linear regressions, we examine relative importance of each factor considered when calculating the wet bulb globe temperature on the spatial variability of its maximum average and minimum average values over the Chicago grids. The linear models can almost perfectly capture the variability in wet bulb globe temperature, with adjusted *R* ^2^ of around 0.99 and 0.97 for the models of maximum and minimum wet bulb globe temperature, respectively. The relative importance is given by the regression coefficients, with the sign of the coefficients showing the direction of the associations (Figure 6). The signs are consistent with Equation 1 of the Methods, with wet bulb globe temperature increasing with air temperature, relative humidity, and solar radiation, and decreasing with wind speed. Across members of the ensemble, air temperature has the greatest role in controlling the spatial variability of both maximum and minimum average wet bulb globe temperature over the city at this scale. The second most important variable depends on whether the variables are scaled before the regression coefficients are computed. For unscaled variables, wind speed is most important, while relative humidity becomes more important when the variables are scaled due to its much larger magnitude compared to wind speed. Solar radiation is generally the least importance, except for minimum average wet bulb globe temperature, which may seem counterintuitive, but is because the minimum wet‐bulb temperature can be right at dawn for some grids with some non‐zero shortwave, which becomes relatively more important when the variables are scaled.

### 3.5 Income‐Based Disparities in Different Metrics of Heat Exposure

**Figure 7:**
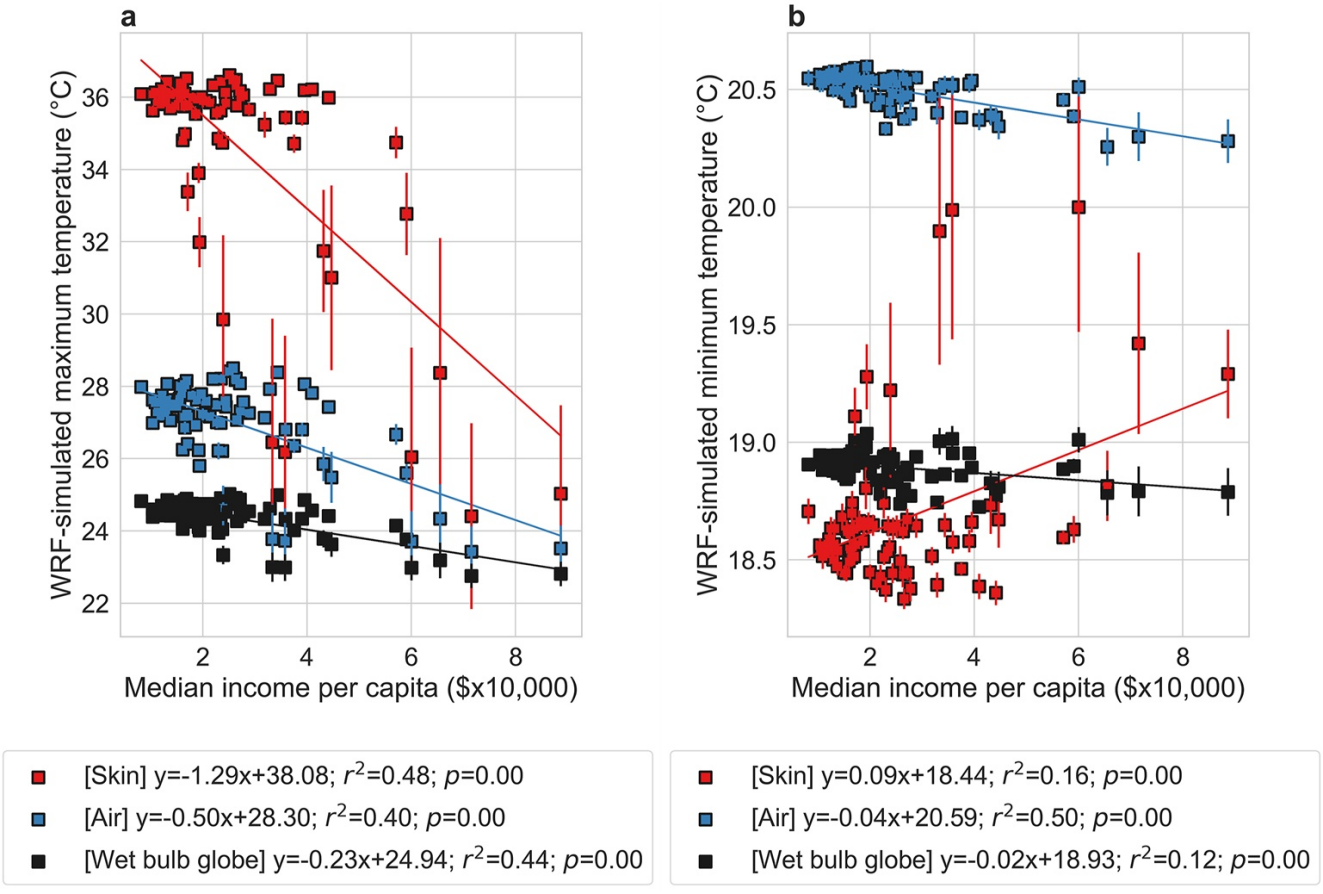
Income‐based disparities in different measures of heat exposure. Associations between WRF simulated (a) maximum and (b) minimum average skin temperature, air temperature, and wet bulb globe temperature in summer 2018 for 77 neighborhoods in Chicago and the corresponding median income per capita. The points represent the mean values from the five members of the ensemble and the error bars correspond to standard errors across the members. The lines of best fit are shown and the associated equations, coefficients of determination, and *p* values are in the legend.

A reason for using neighborhood‐scale summaries from the model simulations is to match them to socioeconomic data collected for the same geographic regions. Several studies have examined disparities in heat exposure in US cities using similar methodology, but focusing on satellite‐derived skin temperature (Chakraborty et al., 2020; Hoffman et al., 2020; Hsu et al., 2021). Since skin temperature is less relevant to physiological response to heat than heat indices that combine multiple factors, we examine associations between skin temperature, air temperature, and wet bulb globe temperature against the median income per capita for Chicago (Figure 7). Overall, we see negative and statistically significant (*p* < 0.05) relationships between income and the maximum summer averages of all these variables, suggesting lower peak heat exposure in richer Chicago neighborhoods (Figure 7a). However, the slope of these relationships is strongest for skin temperature (−1.29°C per $10,000 higher median income per capita) and weakest for wet bulb globe temperature (−0.23°C per $10,000 higher median income per capita). This makes conceptual sense since air and wet bulb globe temperature are strongly affected by advection and the latter is also influenced by urbanization‐induced drying (Chakraborty et al., 2022). Overall, we should be cautious about quantitative estimates of disparities in heat exposure based on skin temperature. For minimum summer averages of temperature, the results show less consistency, with skin temperature showing a positive association with income and wet bulb globe temperature showing almost no association (Figure 7b). The nighttime associations with income are also not entirely consistent across model configurations, which partly relate to how anthropogenic heat is prescribed in WRF (see Section 3.6).

### 3.6 Confirming Key Results With Different Model Configurations

We chose our default model configuration based on previous work done over the Great Lakes region, which was focused on improving prescribed boundary conditions for lake surface temperature and capturing near‐surface climate over the lakes (J. Wang et al., 2022). Due to the sensitivity of urban climate to model complexity (Qian et al., 2022), we consider a couple of other configurations and run them for the control simulation. First, we run a five‐member ensemble using the same configuration but using only the Noah LSM instead of BEP and BEM. Without the multi‐layer urban canopy, the urban land is treated as a slab with modified surface properties with no explicit building impact on airflow or energy exchange. We also run a three‐member ensemble with BEP and BEM, but using the Mellor–Yamada–Janjic (MYJ) scheme (Janjić, 1994), another commonly used boundary layer scheme in mesoscale models, instead of the YSU scheme. Finally, since the 4 km model grid is somewhat coarse, being similar in area to the community areas, we also set up a three‐way nested domain (12–4 km–1.333 km) that can simulate finer‐scale variability to examine consistency of our results, especially relevant for the disparity analysis using neighborhood‐scale socioeconomic data. Only one ensemble member or simulation is done for the nested case due to computational bottlenecks for a full summer simulation.

**Figure 8:**
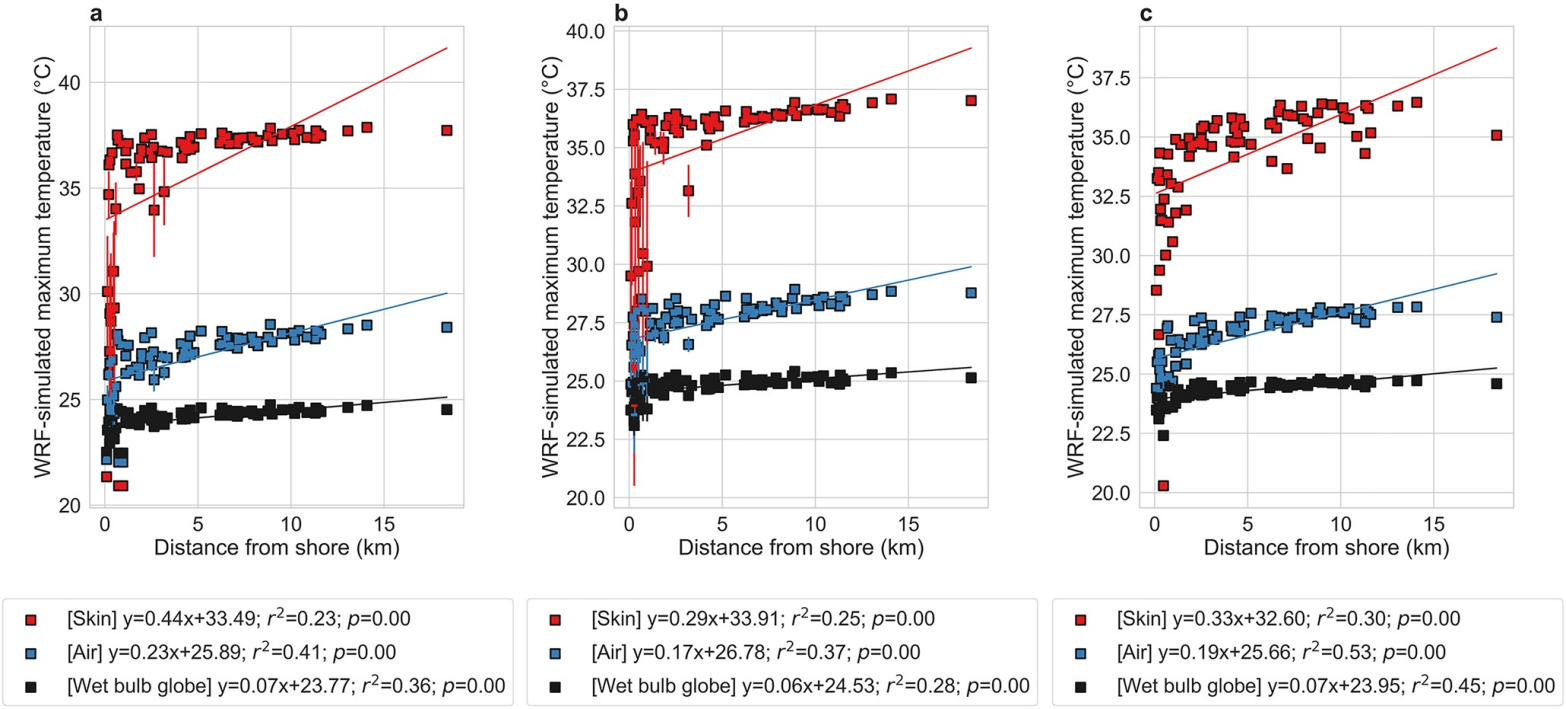
Comparing lake‐to‐land gradients in maximum average of the variables across model configurations. Associations between maximum average skin temperature, air temperature, and wet bulb globe temperature in summer 2018 for Chicago neighborhoods and the corresponding distance of the centroid of each community area to the lake shore from WRF simulations with (a) NOAH land surface model, (b) MYJ boundary layer scheme, and (c) three‐way nesting. The points represent the mean values (five‐member ensemble for (a) and three‐member ensemble for (b) and the error bars correspond to standard errors across the members when they are used. The lines of best fit are shown and the associated equations, coefficients of determination, and *p* values are in the legend.

**Figure 9:**
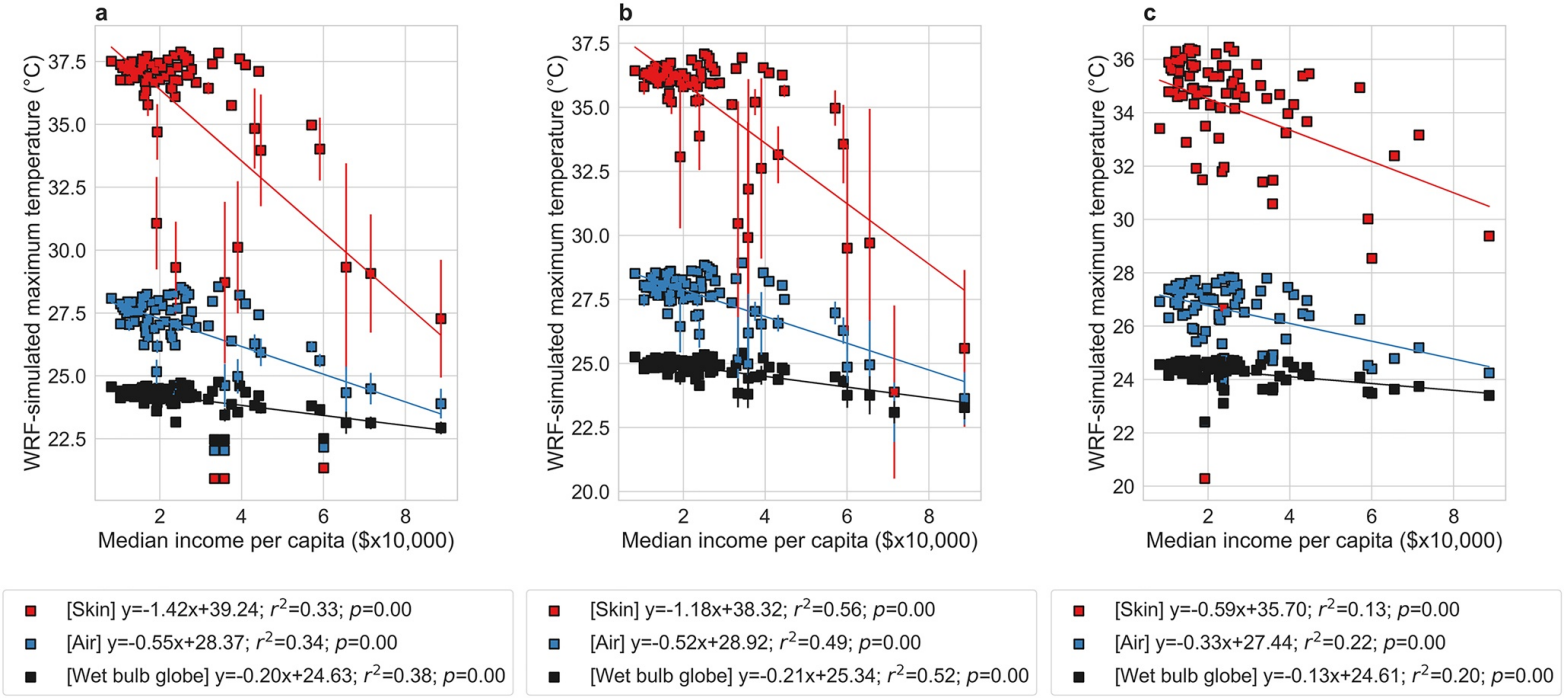
Income‐based disparities in maximum average of the variables across model configurations. Associations between maximum average skin temperature, air temperature, and wet bulb globe temperature in summer 2018 for 77 neighborhoods in Chicago and the corresponding median income per capita from WRF simulations with (a) NOAH land surface model, (b) MYJ boundary layer scheme, and (c) three‐way nesting. The points represent the mean values (five‐member ensemble for a and three‐member ensemble for (b) and the error bars correspond to standard errors across the members when they are used. The lines of best fit are shown and the associated equations, coefficients of determination, and *p* values are in the legend.

We consider the lake‐to‐land gradients in maximum and minimum average skin temperature, air temperature, and wet bulb globe temperature and the associated correlations with median income as the standard results for comparison. In all cases, the maximum average skin temperature, air temperature, and wet bulb globe temperature show a positive correlation with distance from the lake shore, a proxy for the combined impact of the lake and urbanization. More importantly, similar to our main results (Figures 1b and 4), the gradient is steeper for skin temperature than for air temperature, while wet bulb globe temperature shows the least gradient (Figure 8). Although different configurations show different magnitudes of the gradients, which is a function of both model configuration and the internal model variability in a coupled framework, the stronger sensitivity of skin temperature is consistently seen. Similarly, disparities in maximum heat exposure regardless of the measure used are seen across configurations (Figure 9). However, the magnitudes of the disparities are higher for skin temperature than for air temperature, and wet bulb globe temperature shows the least disparity.

For minimum average values, the results are mixed. For lake‐to‐land gradients, the sensitivities are slightly negative or close to zero in our default runs as well as using the MYJ scheme and the three‐nested simulation (Figure 4; Figures S9b and S9c in Supporting Information S1). But the Noah LSM shows positive associations with the distance from the lake shore. This could be because of lack of explicit building effects in Noah LSM compared to the other configurations. Note, however, that the negligible gradient in wet bulb globe temperature is still captured by this configuration (Figure S9a in Supporting Information S1). Overall, for daytime, when heat stress would be maximum, our results show qualitatively consistent results regardless of model setup, physics schemes, and spatial resolutions, with similar lake‐to‐land gradients and associations with median income (Figures 1, 4, 7, 8, 9; Figures S9 and S10 in Supporting Information S1). Moreover, air temperature and heat stress show lower spatial variability than skin temperature, which makes conceptual sense and is in line with previous observational and modeling estimates at various scales (Chakraborty, Newman, et al., 2023; Chakraborty et al., 2022; Ho et al., 2016; Venter et al., 2021). However, we did find that the models configurations can give conflicting results for disparities in minimum average measures of heat exposure (Figure 7; Figure S10 in Supporting Information S1), which requires better representation of nighttime urban processes in the model.

## 4 Discussion

The majority of the global human population lives near water bodies, whether in coastal areas or along freshwater shorelines (Crowell et al., 2007; Kummu et al., 2011). Similarly, large cities have frequently developed along the waterfront, with 14 of 17 global megacities (population >10 million) and around 40% of major cities (population between 1 and 10 million) being in coastal areas (Tibbetts, 2002). In 2010, ∼40% of the US population lived in coastal shoreline counties (adjacent to the open ocean, major estuaries, and the Great Lakes), which had disproportionate long‐term (1970–2010) area‐adjusted population growth (∼3.5 times the nationwide average) (*NOAA National Centers for Environmental Information*, *Climate at a Glance*: *Global Time Series*, *Published October 2021*, *Retrieved on 11 November* 2021). These coastal areas will be major centers of future climate change impacts due to continuous population growth and vulnerability to flood risk, sea level rise, heat stress, and other extreme weather phenomena (Diffenbaugh et al., 2007; Hallegatte et al., 2013; Yin et al., 2009). In Chicago, our model simulations show that the presence of Lake Michigan reduces maximum and minimum heat exposure and heat stress over most neighborhoods during summer, with the effects generally strongest in neighborhoods adjacent to the lake shore. In contrast, urbanization increases most metrics of heat exposure, particularly during nighttime, with these increases intensifying with distance from the lake shore. However, both the model and satellite observations show stronger gradients of skin temperature away from the lake shore than of simulated air and wet bulb globe temperature. This makes sense because air and wet bulb globe temperatures are well‐mixed, reducing thermal gradients compared to the surface, which is strongly constrained by the local surface energy budget. Similarly, the impact of urbanization on maximum average wet bulb globe temperature (Figure 2e), which is a standard metric of heat stress linked to health outcomes, is much lower than that on skin temperature. This is partly due to the compensating effect of humidity on heat stress (Chakraborty et al., 2022; Sarangi et al., 2021), which (humidity) is higher near the lake shore. The reduced variability (compared to skin and air temperature) seen for wet bulb globe temperature is also found for wet‐bulb temperature (Figures S4–S7 in Supporting Information S1; see Section 2), a thermodynamic measure of the moisture content of air, often used in the Earth sciences as a proxy for moist heat stress (Im et al., 2017; Raymond et al., 2020; Sherwood & Huber, 2010). These results are important for contextualizing the usefulness of open water for alleviating heat stress (Theeuwes et al., 2013), and the relevance of quantitative estimates of heat reduction and variability in heat exposure using satellite‐derived skin temperature (Y. Chen et al., 2022; He et al., 2019; Manoli et al., 2019). Overall, studies examining public health implications should be careful when providing magnitude of changes for skin temperature, since these changes are not equivalent to changes in physiological metrics of heat stress (Chakraborty et al., 2022; Li et al., 2023; Turner et al., 2022).

Similarly, when examining disparities in different metrics of heat exposure across neighborhoods in Chicago, we find large differences in the sensitivities to median income. Skin temperature generally shows the strongest sensitivity to income, while wet bulb globe temperature shows the weakest association. This result is replicated when we look at associations between these variables (including wet‐bulb temperature) and the Hardship index, a more comprehensive metric for structural urban inequity (Amdat, 2021) (Figure S7a in Supporting Information S1; also see Section 2). The maximum summer averages of all variables generally increase with Hardship index, with the greatest sensitivity for skin temperature and the least for wet‐bulb temperature (Figure S7 in Supporting Information S1). These results indicate the importance of examining disparities in physiologically relevant estimates of heat stress instead of satellite‐derived skin temperature to accurately quantify urban environmental inequities, which has also been noted in a nationwide study using a much simpler urban modeling framework (Chakraborty, Newman, et al., 2023). Although our study does show that using skin temperature, as done frequently in previous studies, may exaggerate the magnitude of disparities in heat exposure, it is important to stress that public health consequences of weather extremes depend on both exposure and vulnerability (Hsu et al., 2021). For heat stress, poorer populations are more vulnerable even when the exposure is identical. For instance, a working air conditioner, generally less available to lower‐income populations (Romitti et al., 2022), was the strongest protective factor for heat‐related death during the 1999 Chicago heat wave (Naughton et al., 2002).

Although the variability in air temperature due to Lake Michigan is consistent with previous estimates (Conry et al., 2015; J. Yang et al., 2022), the interactions between the lake and urbanization on heat stress and its disparities in Chicago are influenced by air temperature, relative humidity, and wind, with a distinct overall response for seasonal extremes (Table 1). These complexities makes it more difficult to predict future heat stress in Chicago and other coastal cities, with multiple factors coming into play, including additional drying and warming due to urban expansion and increased onshore penetration by lake breezes (Conry et al., 2015). Accurately capturing these competing mechanisms requires not just better representations of cities and lakes in models, but also reasonable estimates of dynamic changes in land cover, including urban greening and irrigation practices, which are still poorly constrained (Krayenhoff et al., 2020; Qian et al., 2020, 2022; Sharma et al., 2020; J. Wang et al., 2022), with currently unknown overall impacts on urban heat stress (Im et al., 2017). As we head toward a warmer, wetter, and more urbanized future (Lewis & Maslin, 2015; W. Wang et al., 2021), it is critical to continue to build these tools that can provide accurate and actionable data for urban climate adaptation and mitigation, especially to help vulnerable waterfront communities (Sharma et al., 2020).

## 5 Conclusion

We examine the role of urbanization and Lake Michigan on different measures of heat exposure in Chicago using ensembles of weather model simulations. Urbanization increases heat exposure, while Lake Michigan decreases it. However, the degree of urban and lake impact on heat exposure depends on the metric or variable used. Skin temperature generally responds the most to urban and lake effects, while a physiologically relevant metric of heat stress like wet bulb globe temperature responds the least. This is because of the compensating impacts of humidity on wet bulb globe temperature. Overall, the competing mechanisms mean that lake‐to‐land gradients in maximum wet bulb globe temperature is much weaker than well‐known gradients for air and skin temperature. Consequently, potential disparities in maximum ambient heat stress are less than what would be seen from satellite observations or in air temperature alone. Our results demonstrate the importance of using appropriate metrics of heat stress to provide relevant quantitative estimates from urban heat monitoring efforts and for informing urban adaptation and mitigation strategies.

## Conflict of Interest

The authors declare no conflicts of interest relevant to this study.

## Supporting information

Supporting Information S1

## Data Availability

The spatial polygons for the community areas and the socioeconomic data were accessed through the Chicago Data Portal (City of Chicago, 2014). All neighborhood‐scale summaries generated in this study can be found here (Chakraborty, Wang, et al., 2023). *Code availability*: The WRF model code is open source and can be accessed at https://github.com/wrf-model/WRF. The Google Earth Engine scripts for summarizing WRF simulations and satellite observations can be found through Zenodo (Chakraborty, Wang, et al., 2023).
