# Supplementary material for: Urban Versus Lake Impacts on Heat Stress and Its Disparities in a Shoreline City
Source: Geohealth. 2023 Nov 22;7(11):e2023GH000869. doi: 10.1029/2023GH000869 (PMC10664081; doi:10.1029/2023GH000869)
Supplement: Supplementary file 1 — Supporting Information S1 [file GH2-7-e2023GH000869-s001.docx]

**Supplementary Materials: Urban versus lake impacts on heat stress and its disparities in a shoreline city**

TC Chakraborty^1*^, Jiali Wang^2^, Yun Qian^1^, William Pringle^2^, Zhao Yang^1^, and Pengfei Xue^2,3^

^1^ Atmospheric Sciences and Global Change Division, Pacific Northwest National Laboratory, Richland, WA 99352, USA

^2^ Environmental Science Division, Argonne National Laboratory, 9700 Cass Avenue, Lemont, IL 60439, USA

^3^ Department of Civil, Environmental and Geospatial Engineering, Michigan Technological University, Houghton, MI 49931, USA

**Table of Contents:**

Figures S1 to S10

**
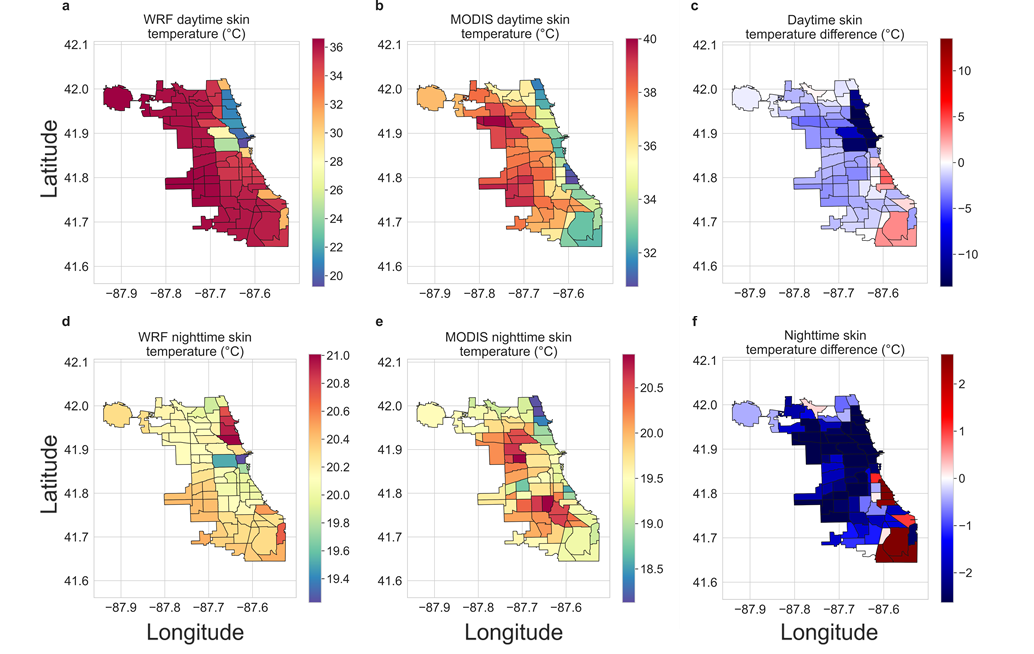
**

**Fig S1.** Spatial patterns of satellite-observed versus simulated skin temperature. Neighborhood-scale summaries of WRF simulated skin temperature corresponding to the MODIS Aqua **a** daytime (~1:30 pm local time) and **d** nighttime (~1:30 am local time) overpass and the MODIS observed daytime and nighttime skin temperature (**b**, **e**, respectively) for summer 2018 in Chicago. **c**, **f** Skin temperature differences between WRF and MODIS (WRF-MODIS) for the neighborhoods.


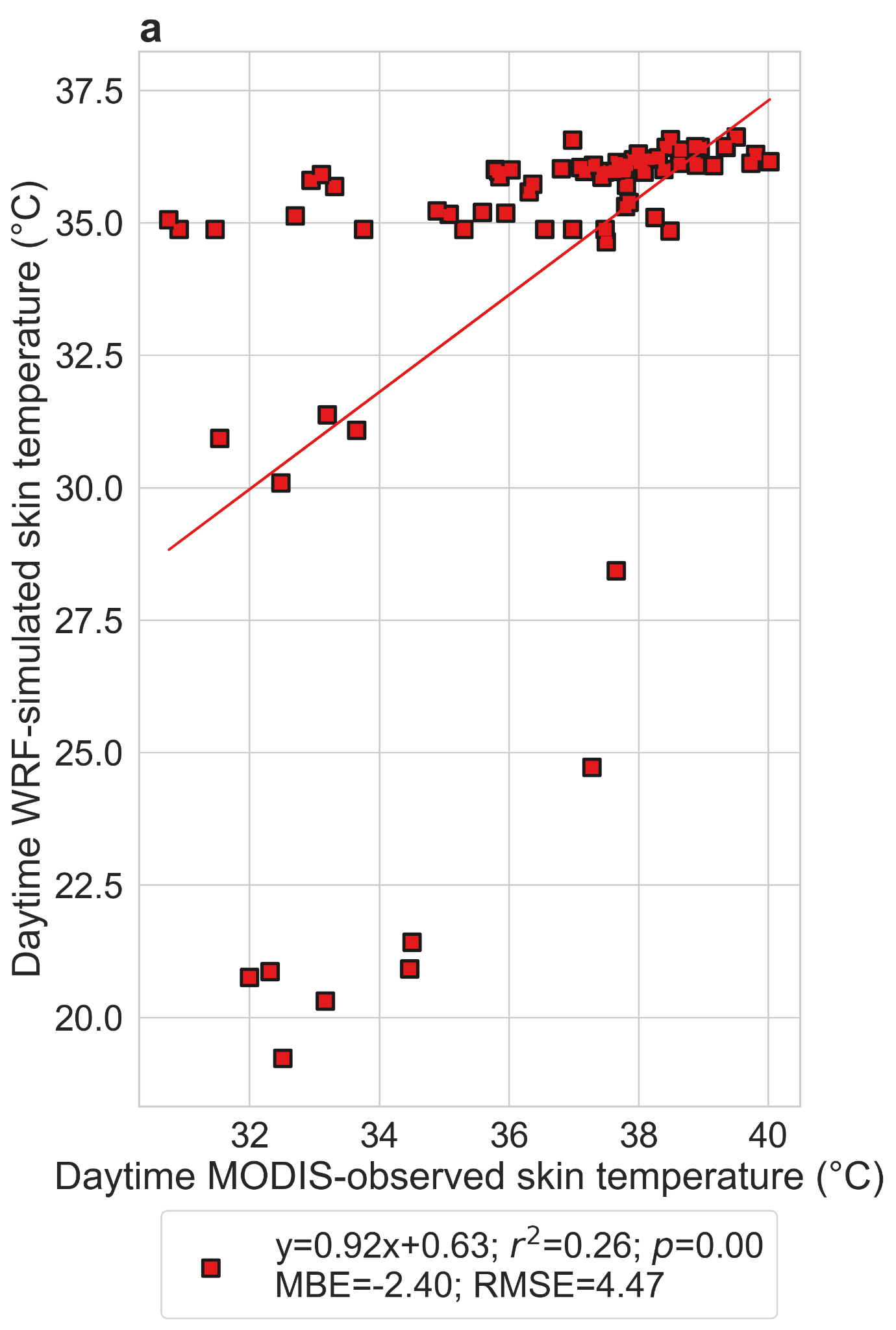

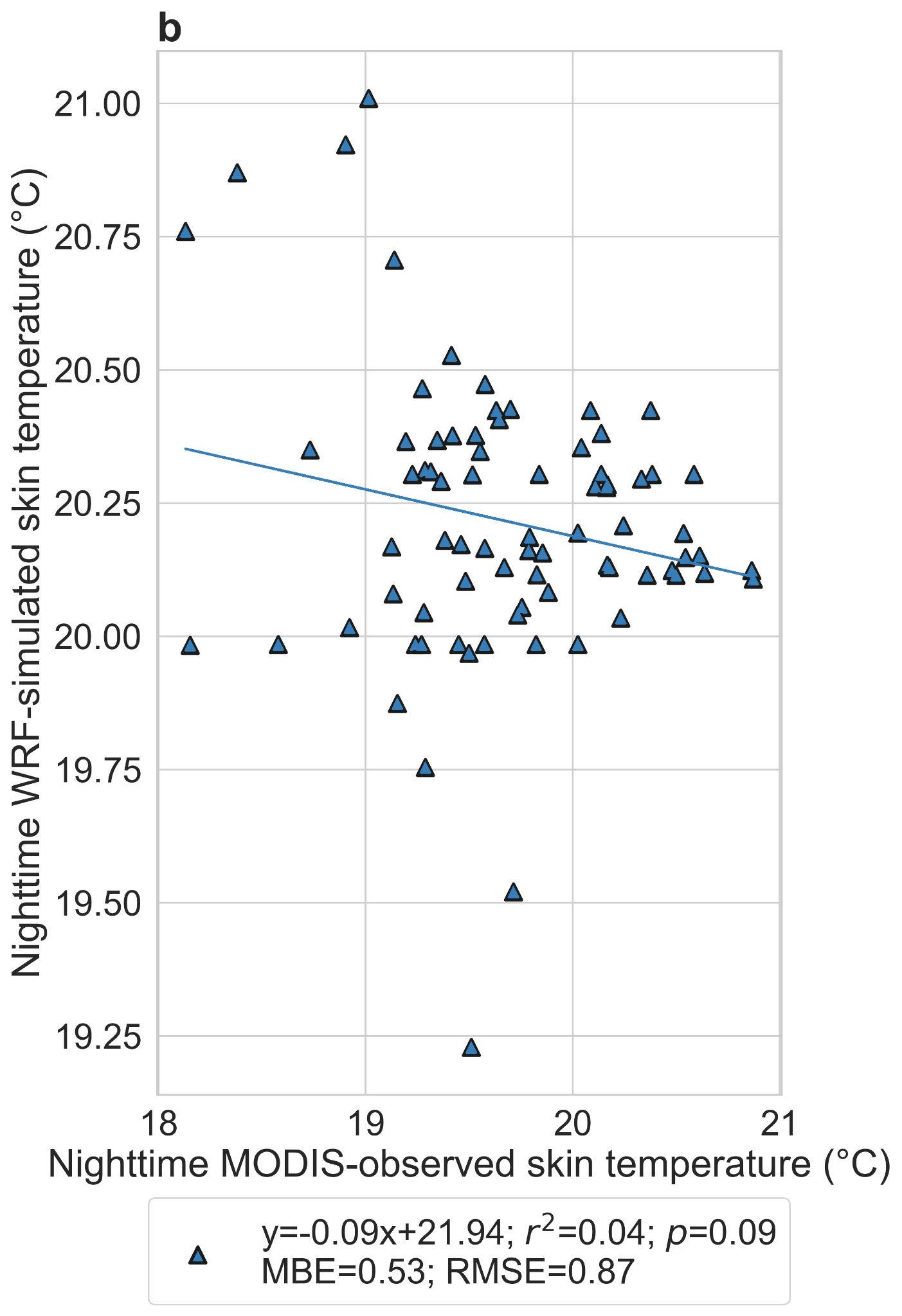

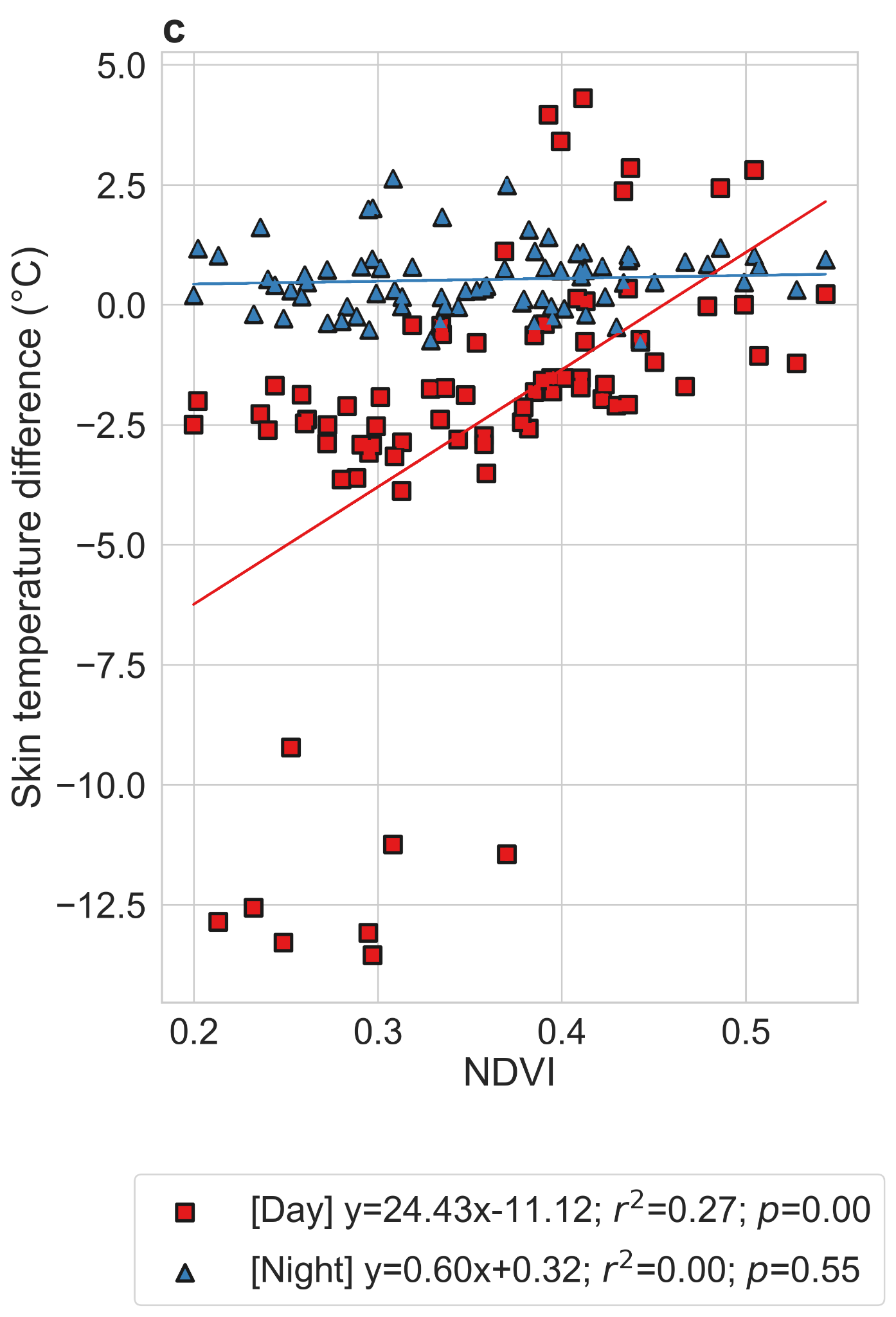


**Fig S2.** Evaluating simulated skin temperature against satellite observations. Associations between WRF simulated and MODIS-observed **a** daytime (~1:30 pm local time) and **b** minimum (~1:30 am local time) skin temperature in summer 2018 for Chicago neighborhoods. **c** Difference between WRF and MODIS skin temperatures (daytime and nighttime) as a function of the summertime neighborhood-mean normalized difference vegetation index (NDVI). The simulated values represent the five-member ensemble mean values from WRF. The lines of best fit are shown and the associated equations, coefficients of determination, and p values are in the legend. The mean bias error (MBE) and the root mean squared error (RMSE) are also noted.


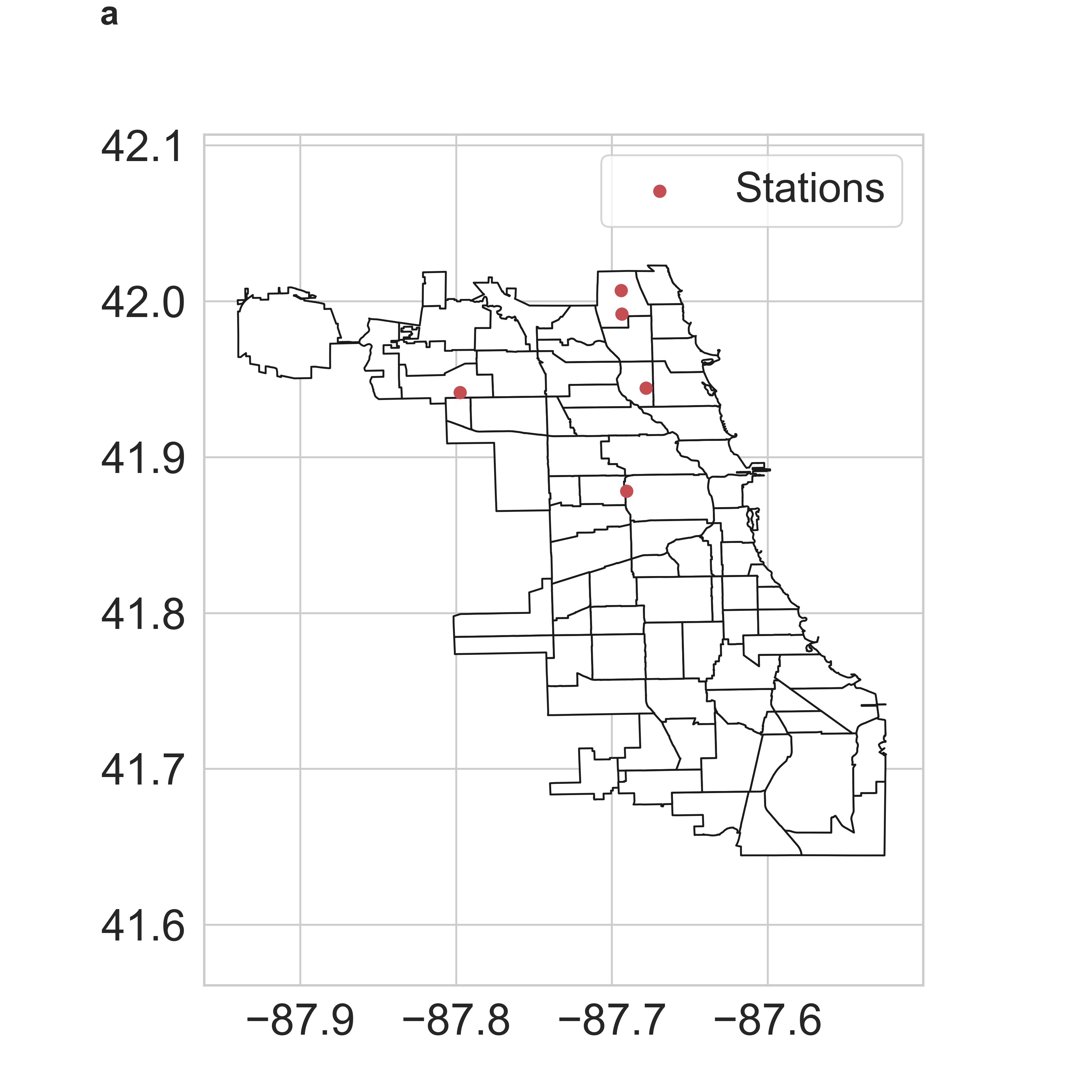

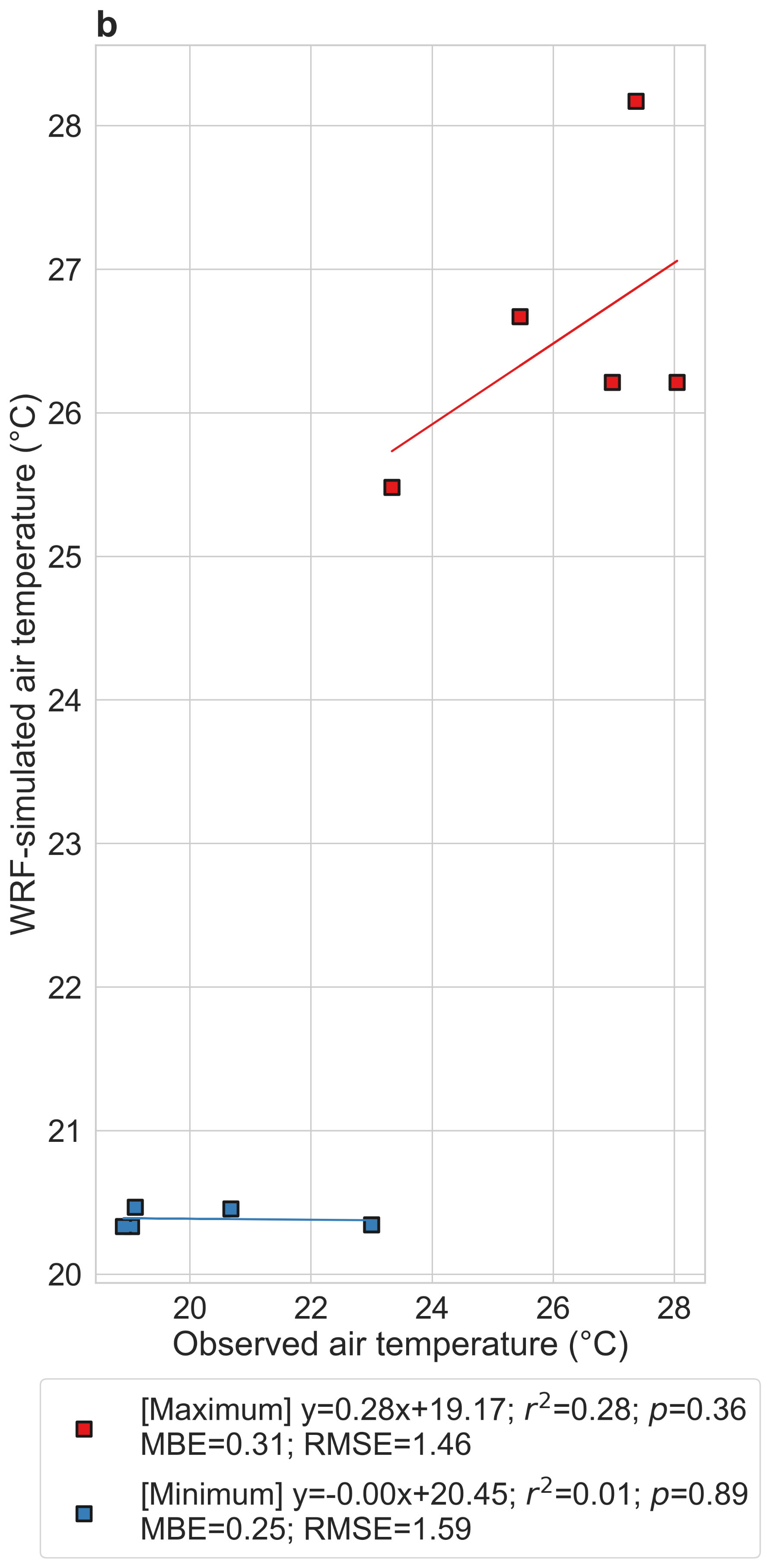

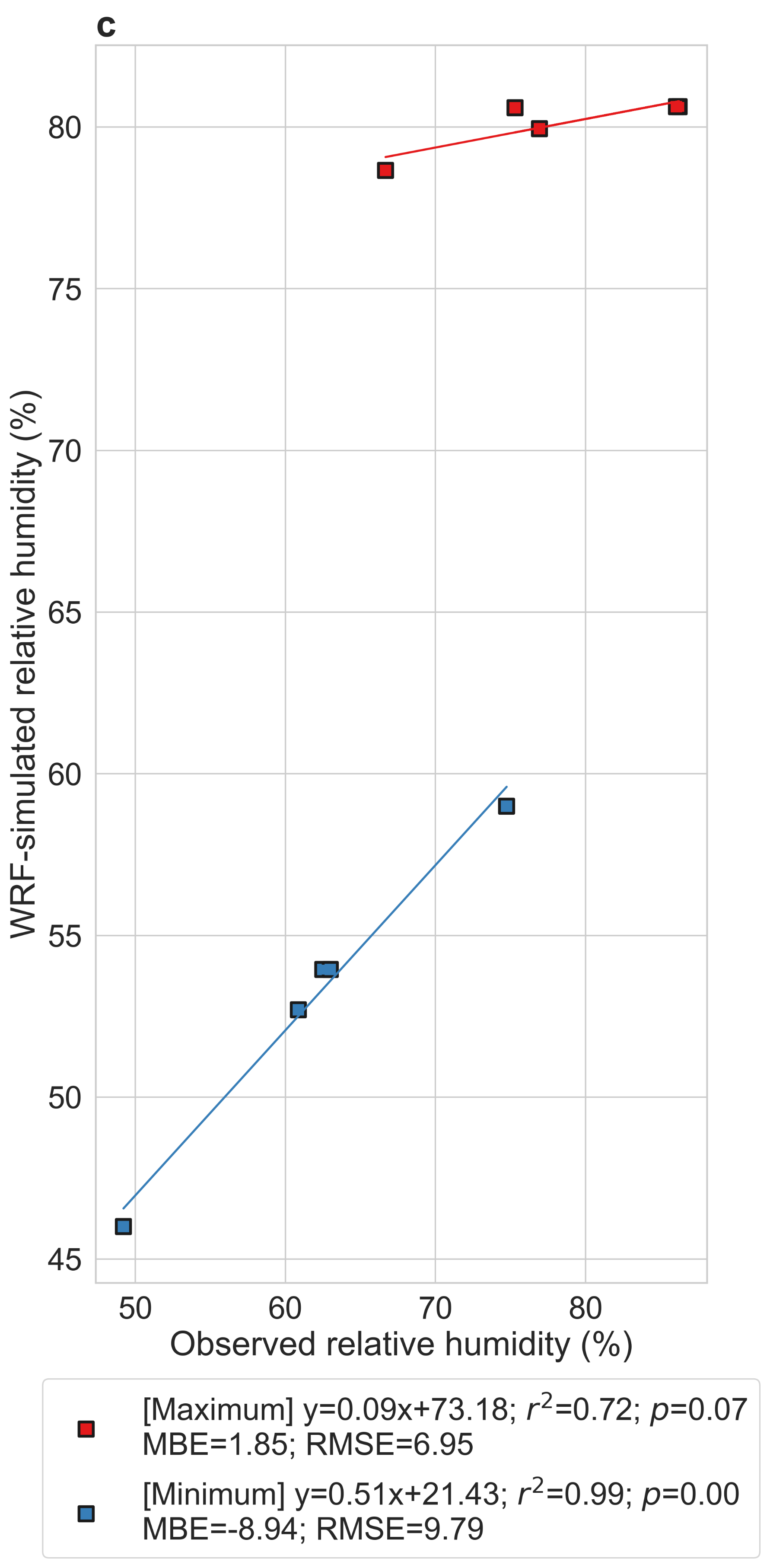


**Fig S3.** Evaluating simulated air temperature and relative humidity against weather station observations. **a** All Netatmo citizen science weather stations within Chicago with complete coverage for summer 2018. Associations between WRF simulated and station-observed maximum and minimum **b** air temperature and **c** relative humidity. The simulated values represent the corresponding neighborhood-level estimates from the five-member ensemble mean values from WRF. The lines of best fit are shown and the associated equations, coefficients of determination, and p values are in the legend. The mean bias error (MBE) and the root mean squared error (RMSE) are also noted.

**
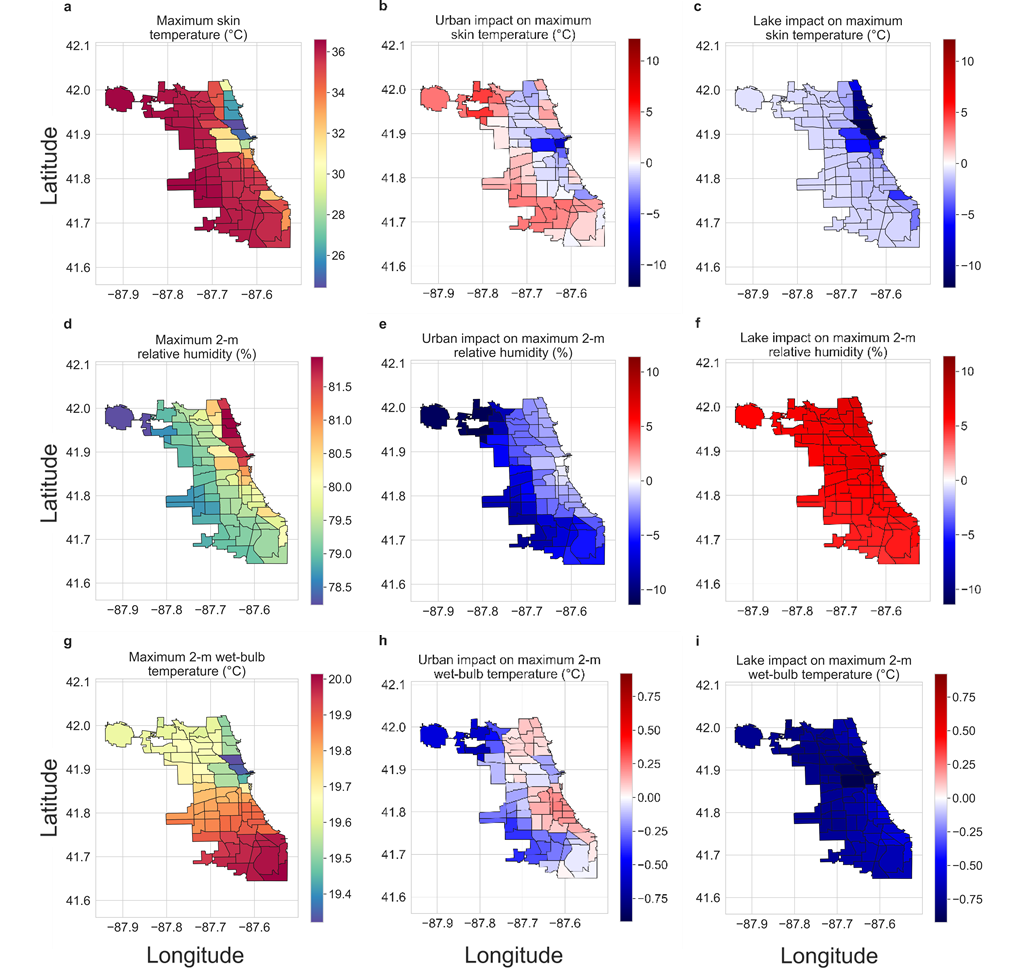
**

**Fig S4.** Urban and lake impacts on other maximum exposure measures. Neighborhood-scale summaries of WRF simulated maximum average **a** skin temperature, **d** relative humidity, and **g** wet-bulb temperature for summer 2018 in Chicago. **b**, **e**, **h** Urban impact on maximum average skin temperature, relative humidity, and wet-bulb temperature, respectively. **c**, **f**, **i** Lake impact on those variables.

**
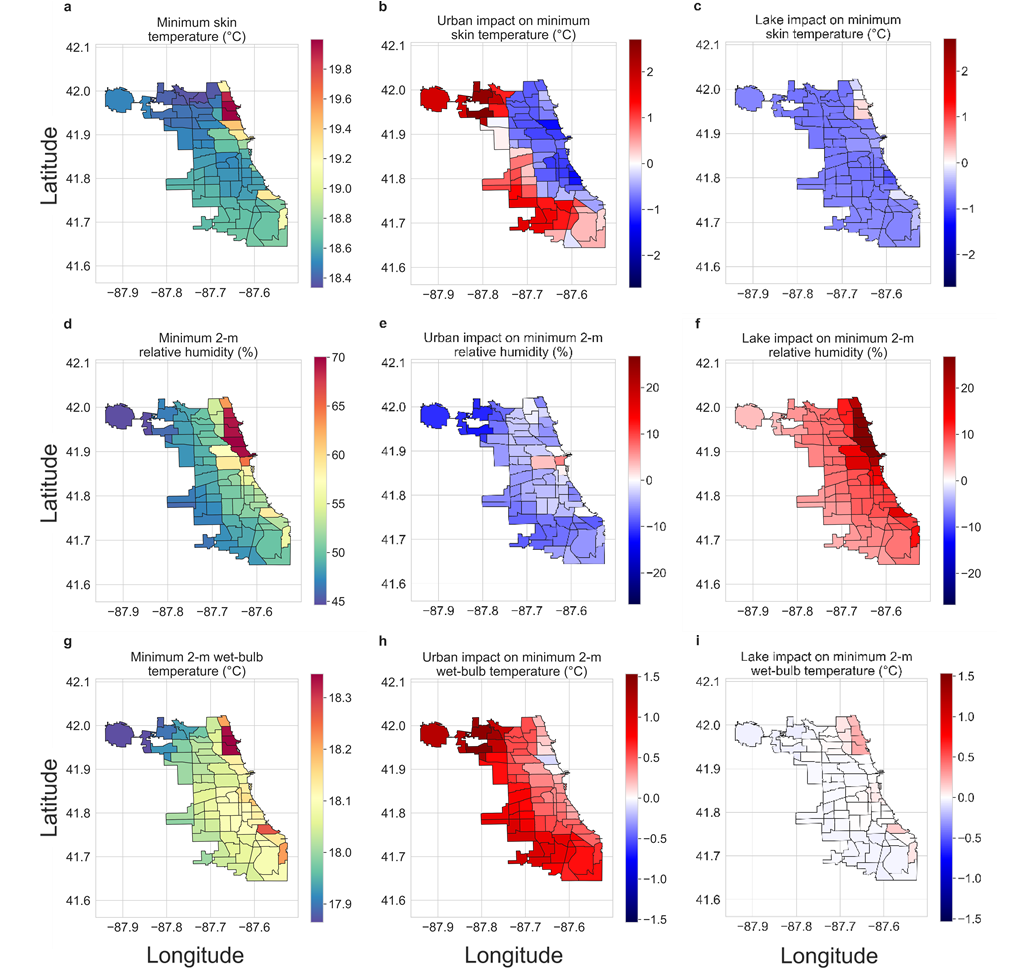
**

**Fig S5.** Urban and lake impacts on other minimum exposure measures. Neighborhood-scale summaries of WRF simulated minimum average **a** skin temperature, **d** relative humidity, and **g** wet-bulb temperature for summer 2018 in Chicago. **b**, **e**, **h** Urban impact on minimum average skin temperature, relative humidity, and wet-bulb temperature, respectively. **c**, **f**, **i** Lake impact on those variables.


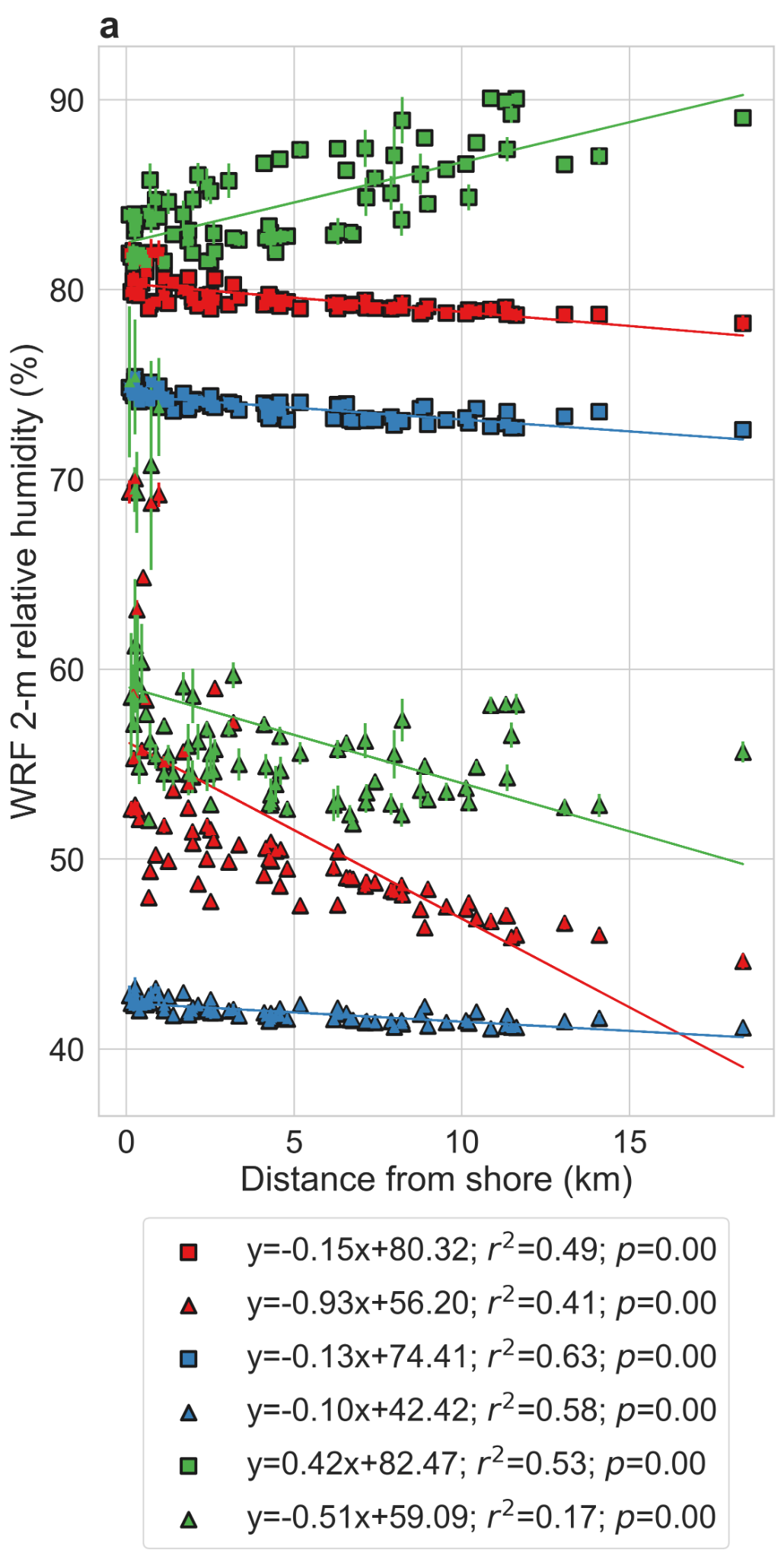

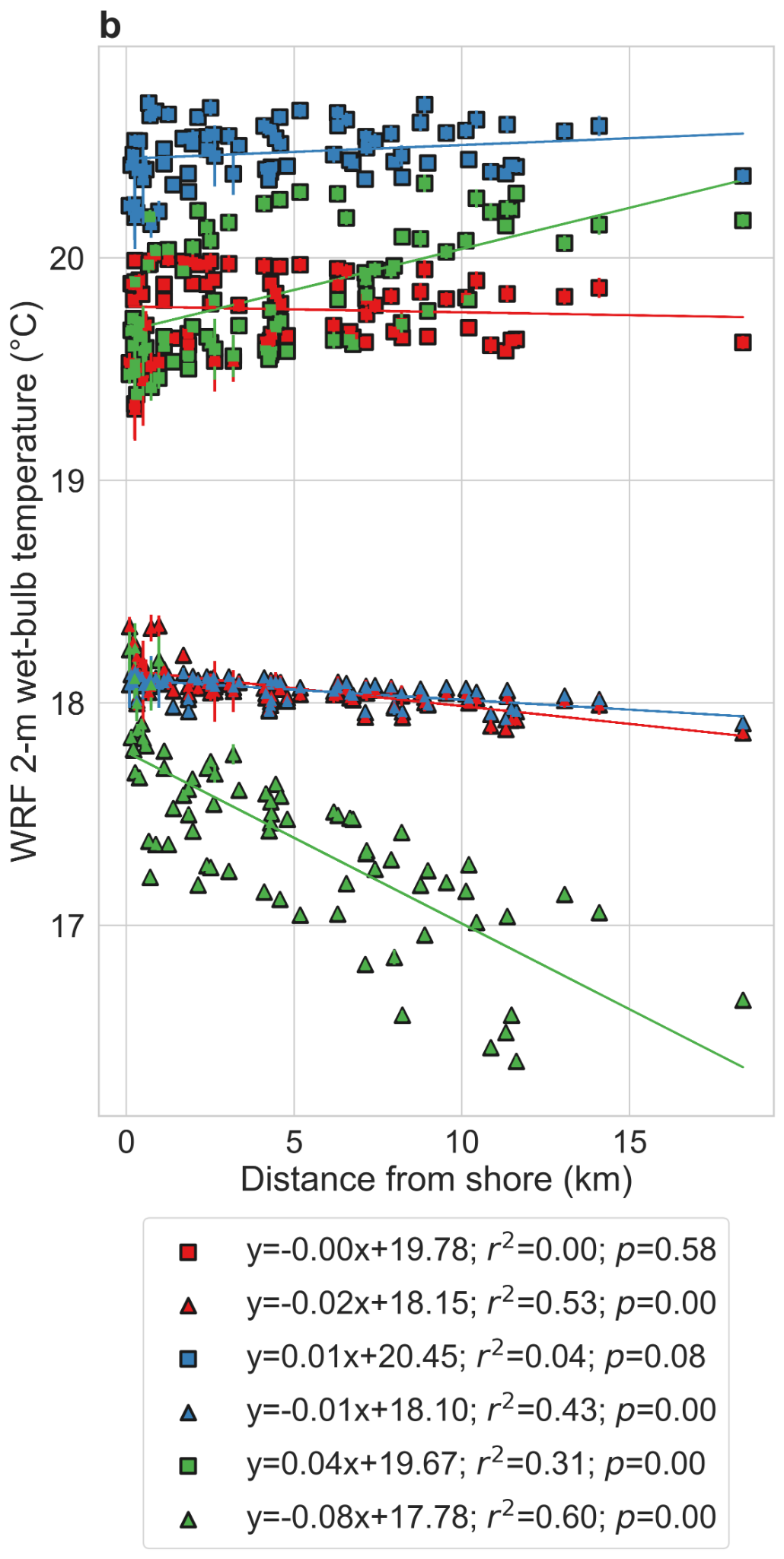

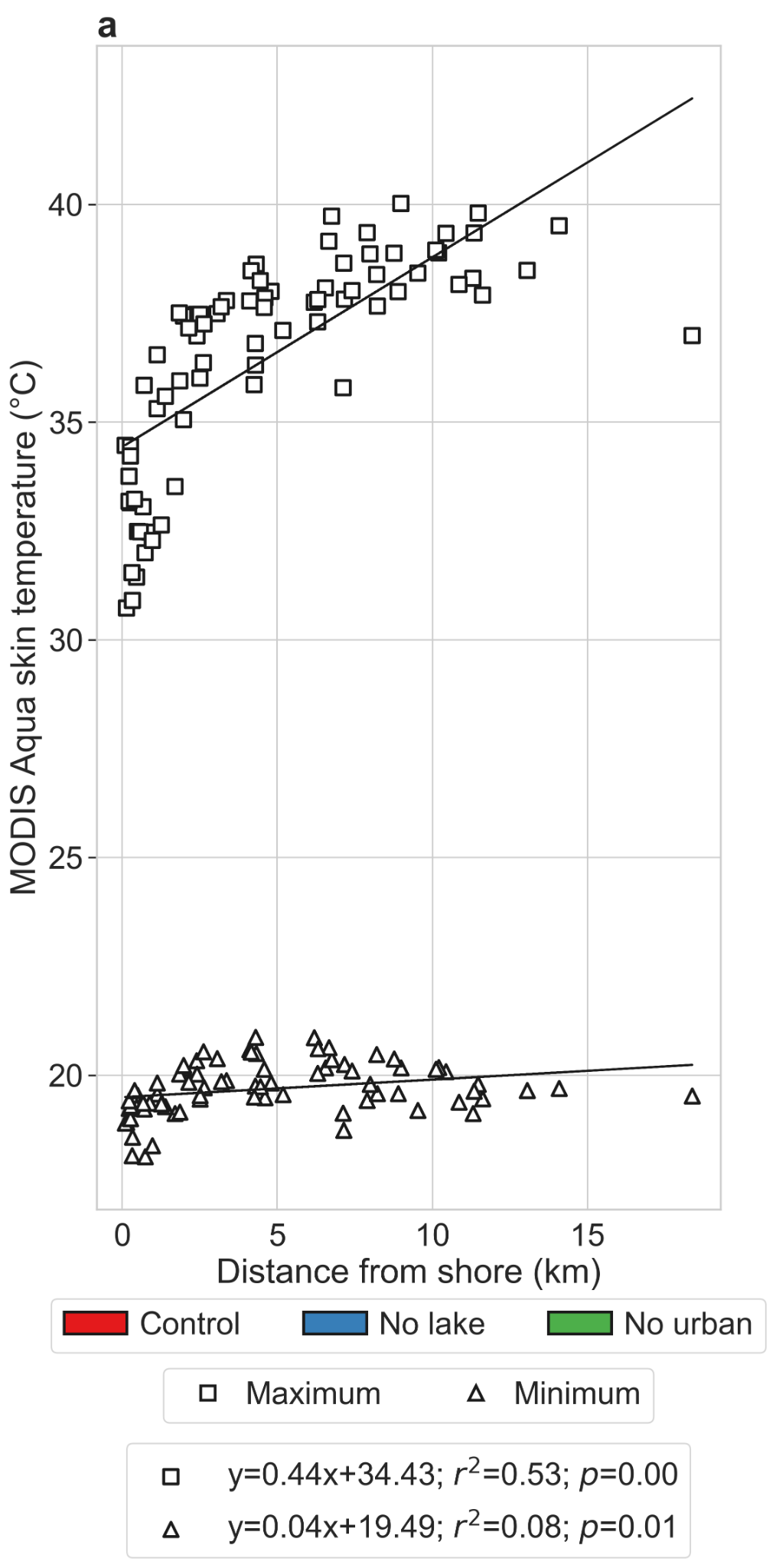


**Fig S6. Lake-to-land gradients of wet-bulb temperature and relative humidity.** Associations between WRF simulated maximum and minimum average **a** relative humidity and **b** wet-bulb temperature in summer 2018 for Chicago neighborhoods and the corresponding distance of the centroid of each community area to the lake shore. The points represent the mean values from the five members of the ensemble and the error bars correspond to standard errors across the members. The lines of best fit are shown and the associated equations, coefficients of determination, and p values are in the legend. The square symbols show the maximum averages and the triangles are for the minimum averages. The three colors (red, blue, and green) show the results for control, no lake, and no urban simulations, respectively.


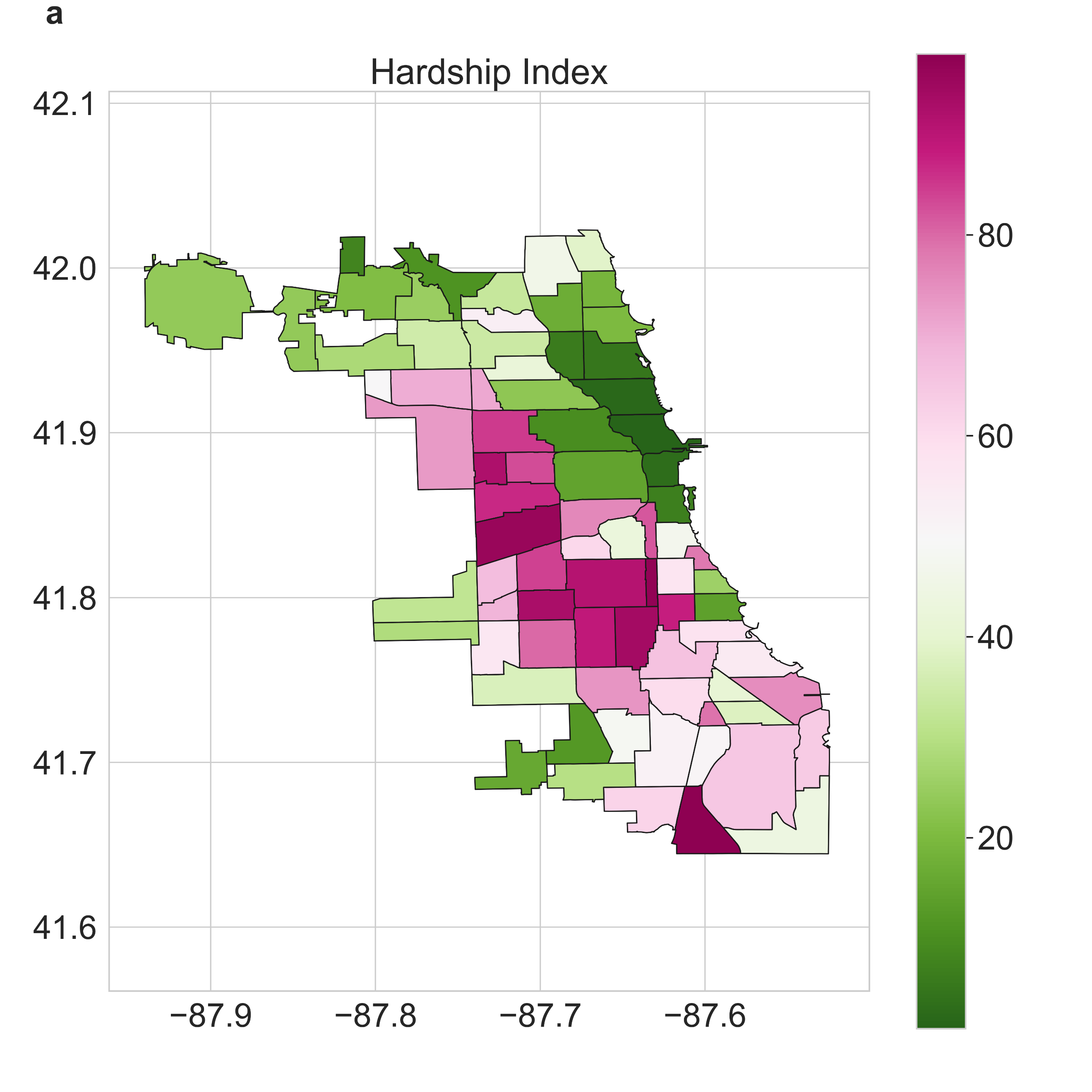

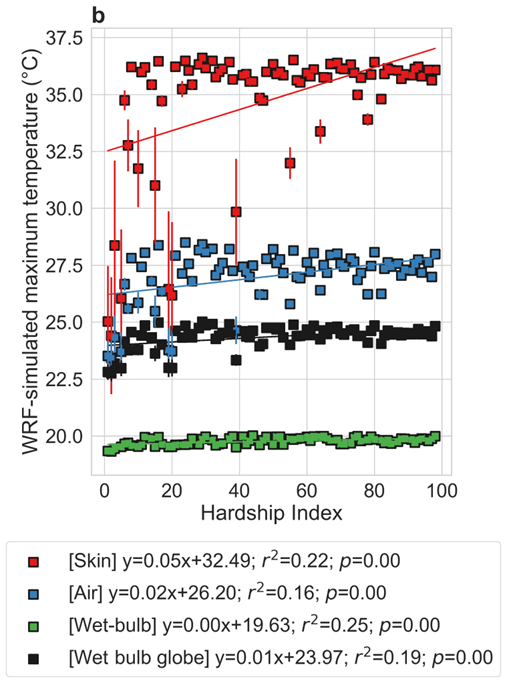

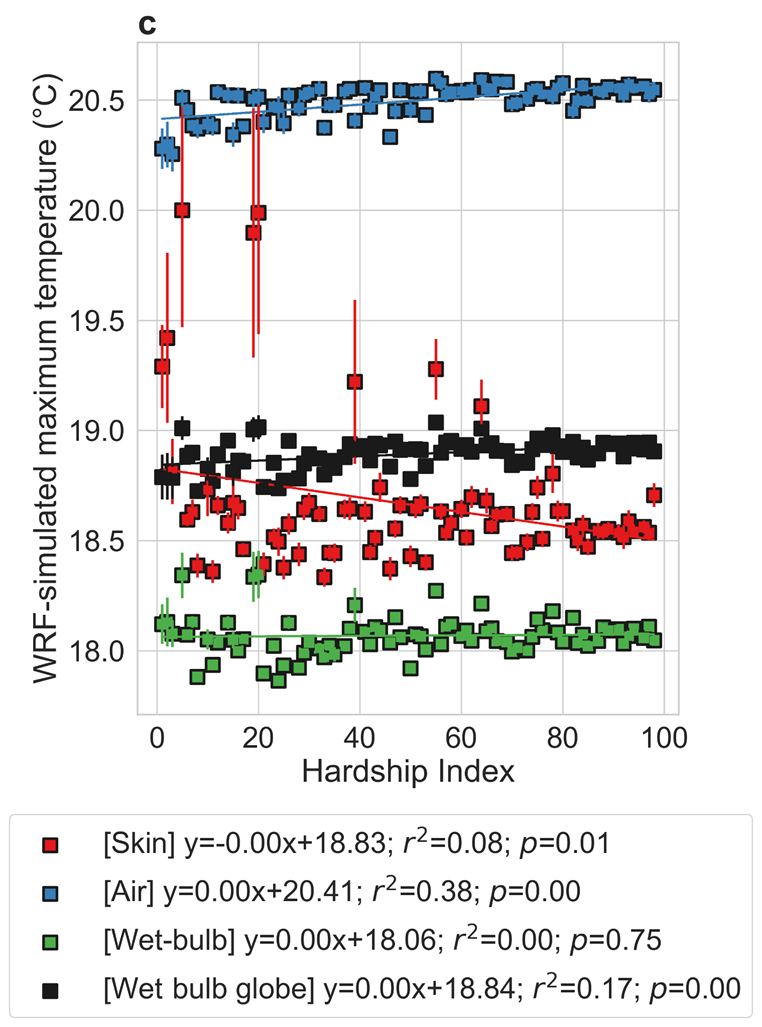


**Fig S7.** Associations between simulated heat exposure measures and Hardship index**. a** Neighborhood-scale summaries of Hardship index. **b** and **c** show associations between WRF simulated maximum and minimum average (respectively) skin temperature, air temperature, wet-bulb temperature, and wet bulb globe temperature in summer 2018 for Chicago neighborhoods and the corresponding Hardship index. The points represent the mean values from the five members of the ensemble and the error bars correspond to standard errors across the members. The lines of best fit are shown and the associated equations, coefficients of determination, and p values are in the legend.


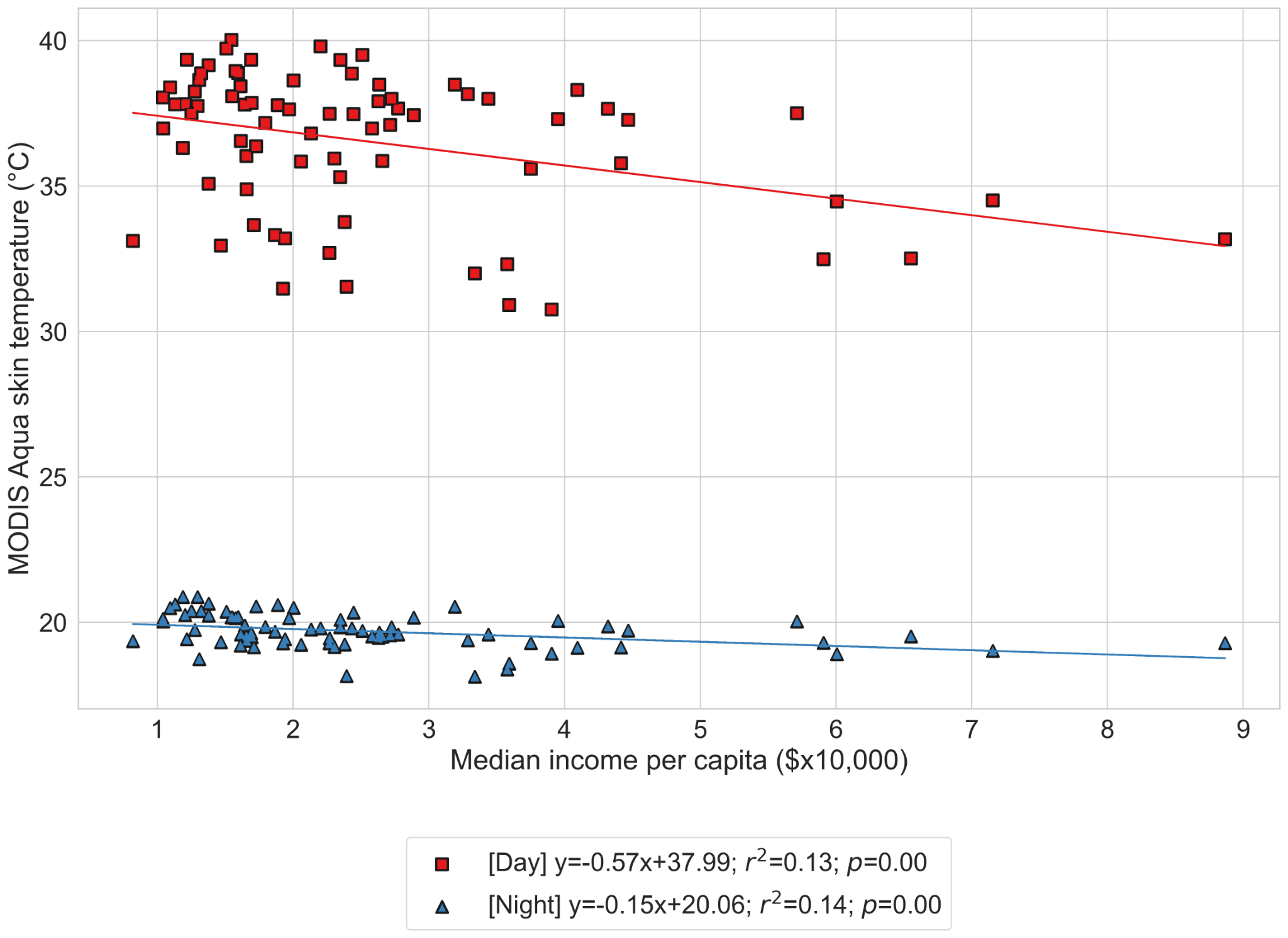


**Fig S8.** Satellite-observed skin temperature versus neighborhood income**.** Associations between MODIS Aqua daytime and nighttime skin temperature for Chicago neighborhoods and the corresponding median income per capita for summer 2018. The lines of best fit are shown and the associated equations, coefficients of determination, and p values are in the legend.


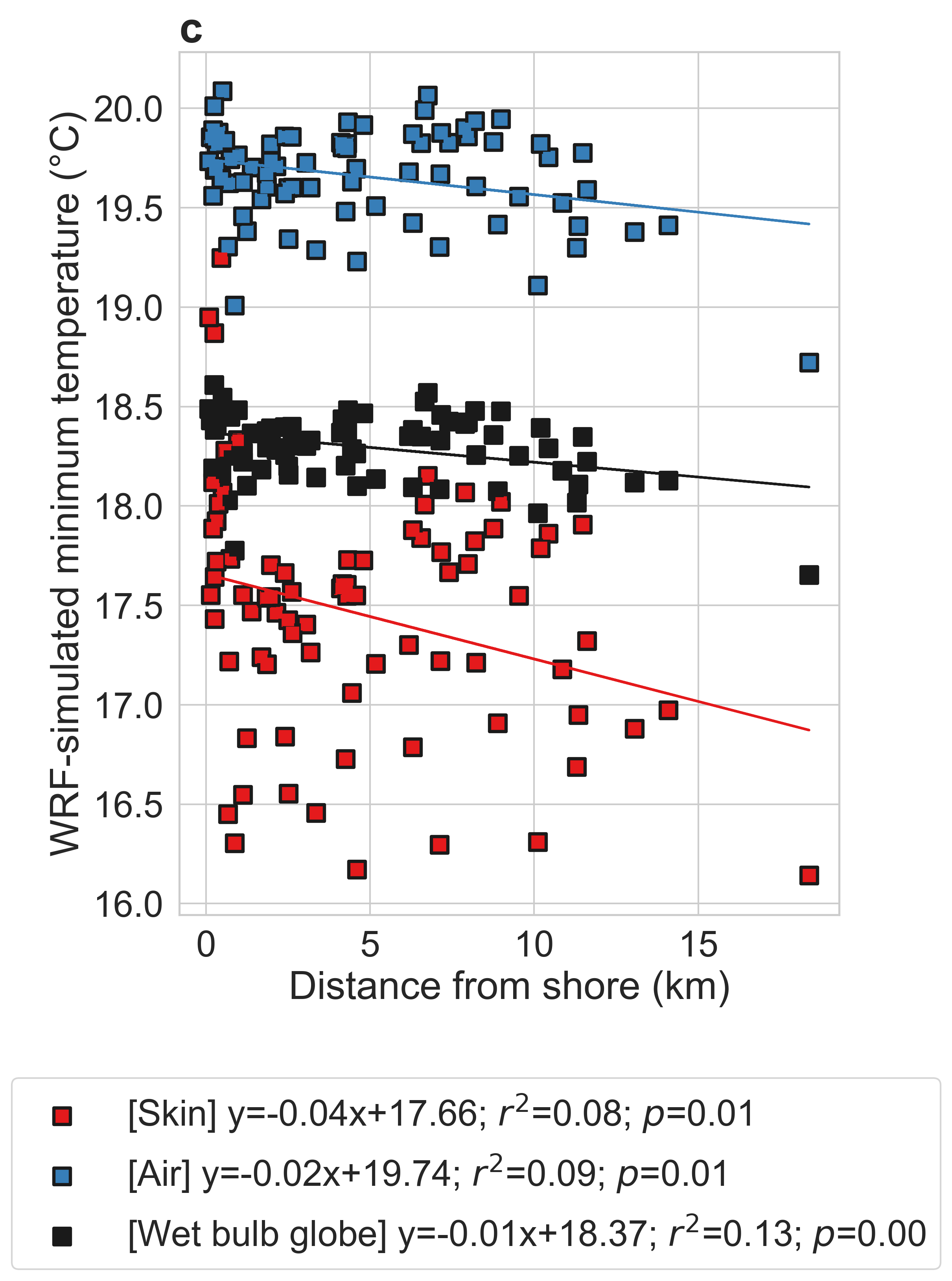

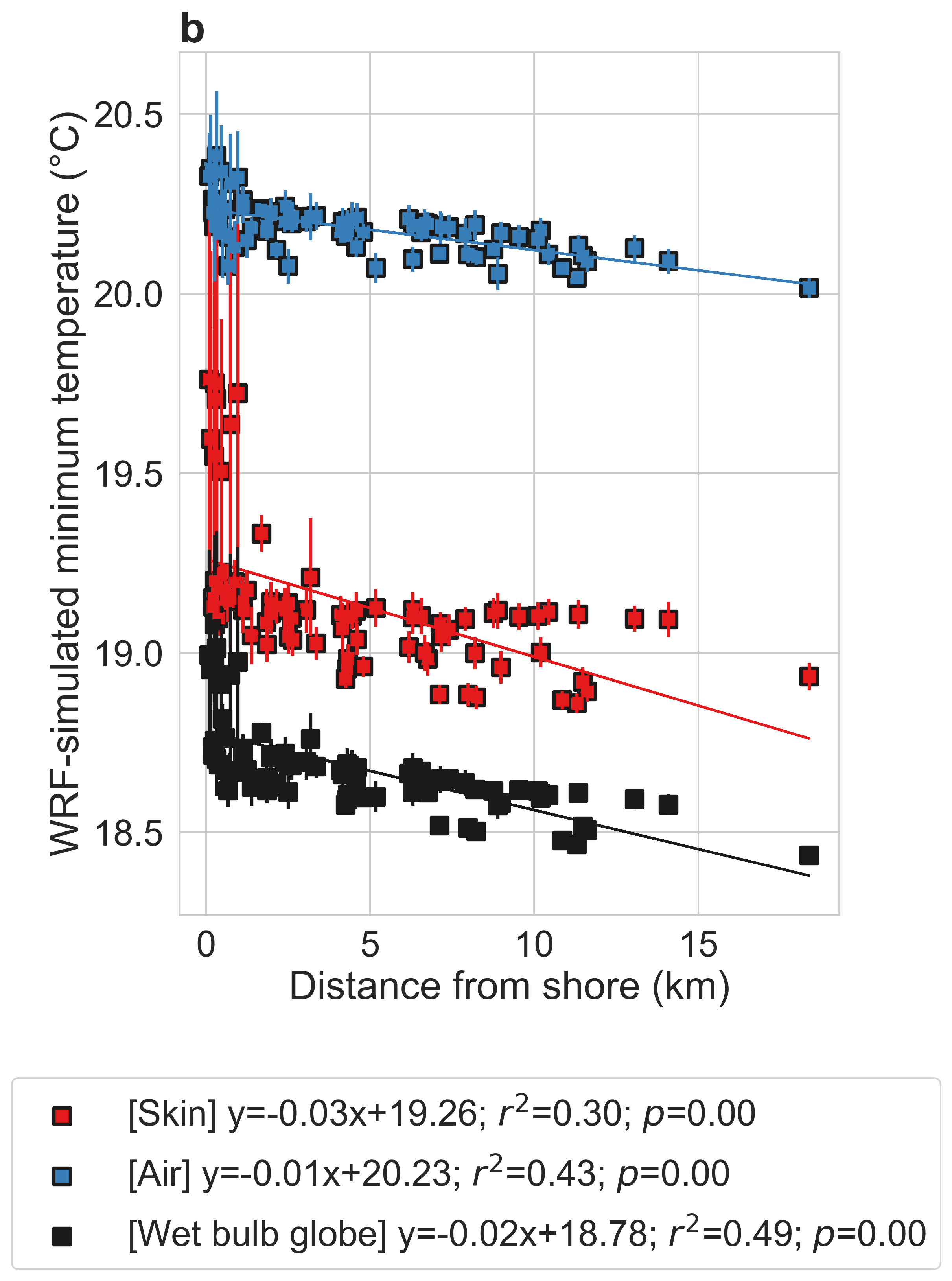

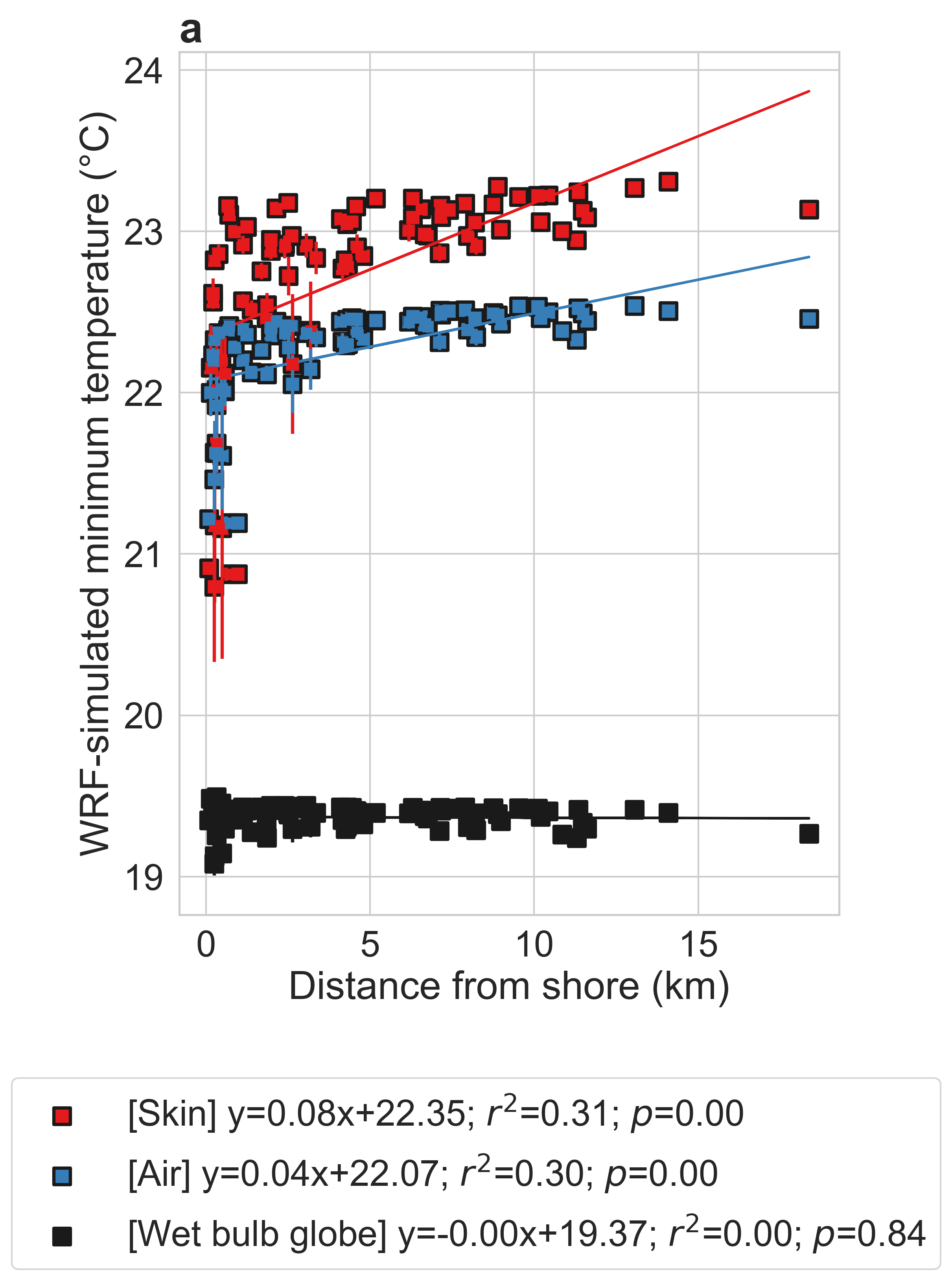


**Fig S9.** Comparing lake-to-land gradients in minimum average of the variables across model configurations**.** Associations between minimum average skin temperature, air temperature, and wet bulb globe temperature in summer 2018 for Chicago neighborhoods and the corresponding distance of the centroid of each community area to the lake shore from WRF simulations with **a** NOAH land surface model, **b** MYJ boundary layer scheme, and **c** three-way nesting. The points represent the mean values (five-member ensemble for **a** and three-member ensemble for **b**) and the error bars correspond to standard errors across the members when they are used. The lines of best fit are shown and the associated equations, coefficients of determination, and p values are in the legend.


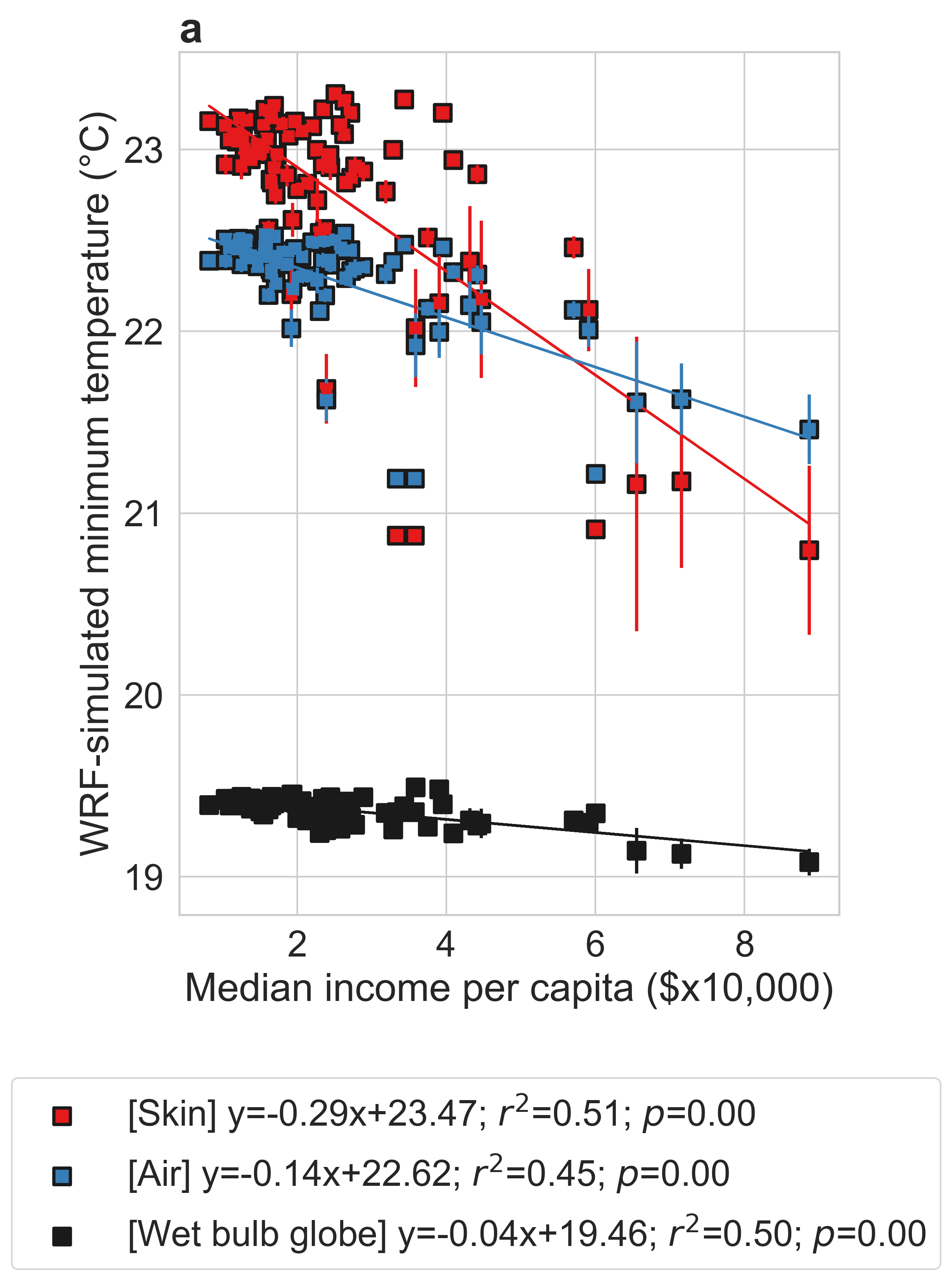

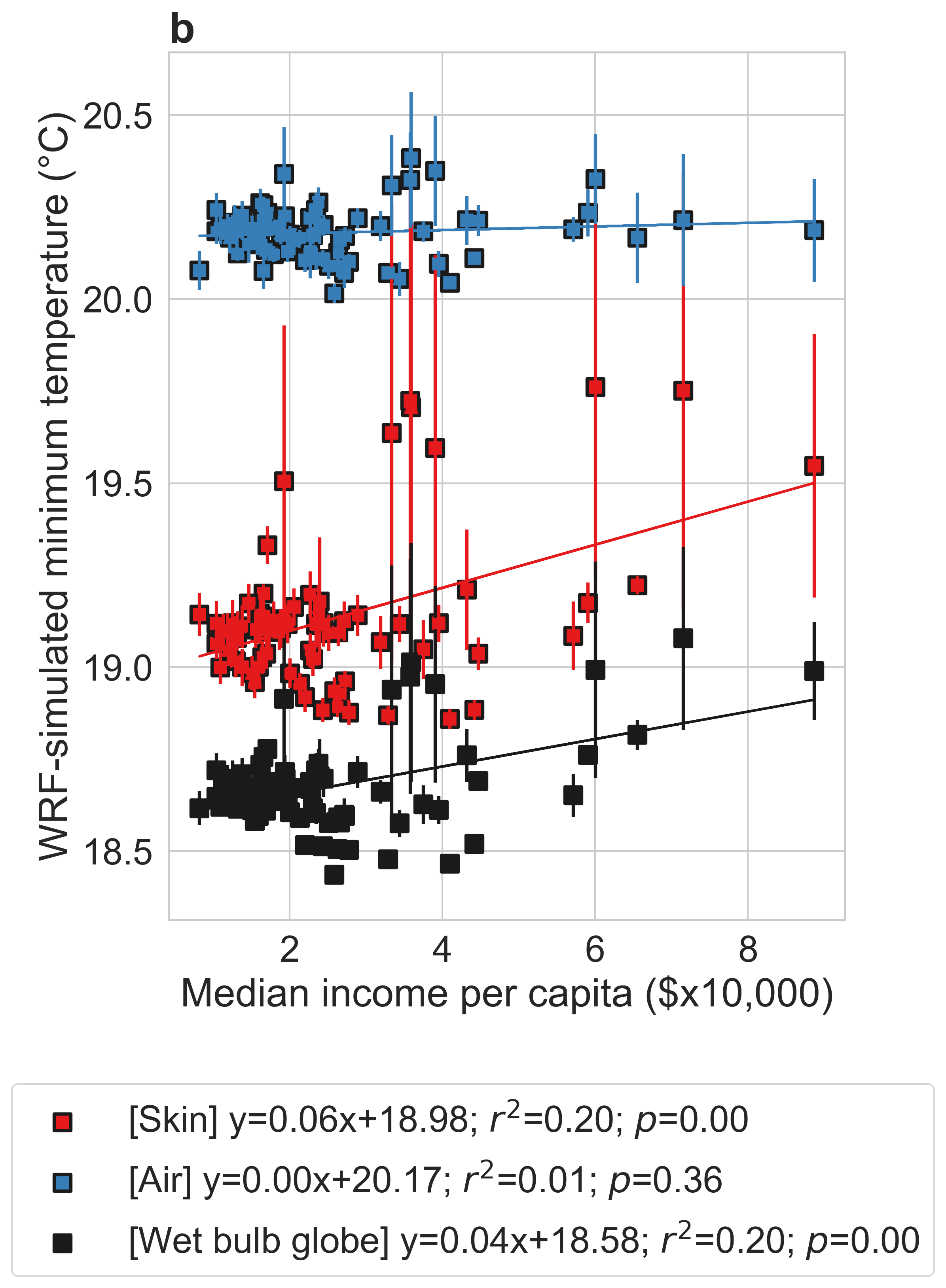

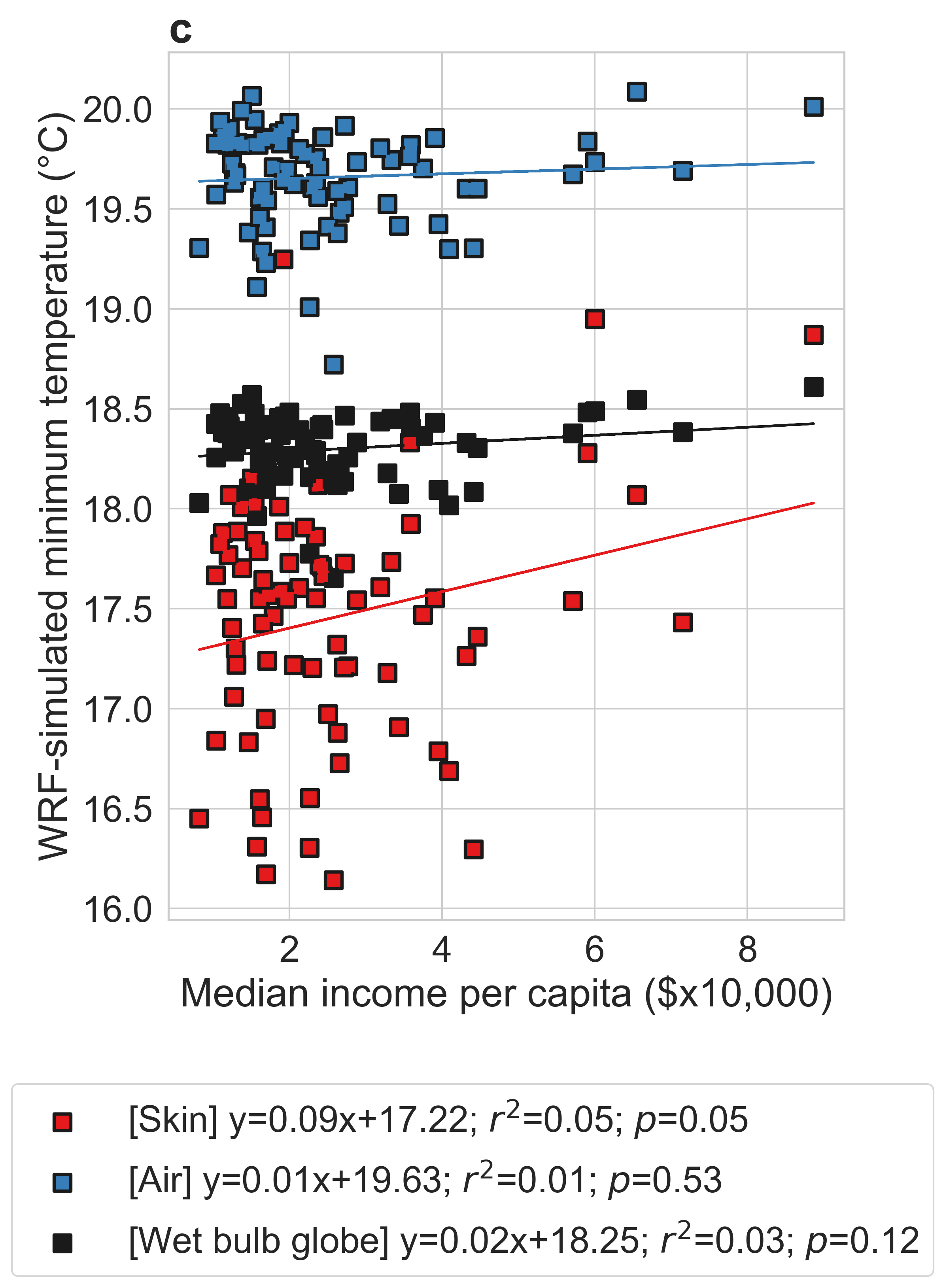


**Fig S10.** Income-based disparities in minimum average of the variables across model configurations. Associations between minimum average skin temperature, air temperature, and wet bulb globe temperature in summer 2018 for 77 neighborhoods in Chicago and the corresponding median income per capita from WRF simulations with **a** NOAH land surface model, **b** MYJ boundary layer scheme, and **c** three-way nesting. The points represent the mean values (five-member ensemble for **a** and three-member ensemble for **b**) and the error bars correspond to standard errors across the members when they are used. The lines of best fit are shown and the associated equations, coefficients of determination, and p values are in the legend.
